# Cell Lineage Analysis of the Mammalian Female Germline

**DOI:** 10.1371/journal.pgen.1002477

**Published:** 2012-02-23

**Authors:** Yitzhak Reizel, Shalev Itzkovitz, Rivka Adar, Judith Elbaz, Adrian Jinich, Noa Chapal-Ilani, Yosef E. Maruvka, Nava Nevo, Zipora Marx, Inna Horovitz, Adam Wasserstrom, Avi Mayo, Irena Shur, Dafna Benayahu, Karl Skorecki, Eran Segal, Nava Dekel, Ehud Shapiro

**Affiliations:** 1 Department of Biological Regulation, Weizmann Institute of Science, Rehovot, Israel; 2 Department of Computer Science and Applied Mathematics, Weizmann Institute of Science, Rehovot, Israel; 3 Department of Biological Chemistry, Weizmann Institute of Science, Rehovot, Israel; 4 Department of Molecular Cell Biology, Weizmann Institute of Science, Rehovot, Israel; 5 Department of Cell and Developmental Biology, Sackler School of Medicine, Tel-Aviv University, Tel-Aviv, Israel; 6 Rappaport Faculty of Medicine and Research Institute, Technion and Rambam Medical Center, Haifa, Israel; Stanford University School of Medicine, United States of America

## Abstract

Fundamental aspects of embryonic and post-natal development, including maintenance of the mammalian female germline, are largely unknown. Here we employ a retrospective, phylogenetic-based method for reconstructing cell lineage trees utilizing somatic mutations accumulated in microsatellites, to study female germline dynamics in mice. Reconstructed cell lineage trees can be used to estimate lineage relationships between different cell types, as well as cell depth (number of cell divisions since the zygote). We show that, in the reconstructed mouse cell lineage trees, oocytes form clusters that are separate from hematopoietic and mesenchymal stem cells, both in young and old mice, indicating that these populations belong to distinct lineages. Furthermore, while cumulus cells sampled from different ovarian follicles are distinctly clustered on the reconstructed trees, oocytes from the left and right ovaries are not, suggesting a mixing of their progenitor pools. We also observed an increase in oocyte depth with mouse age, which can be explained either by depth-guided selection of oocytes for ovulation or by post-natal renewal. Overall, our study sheds light on substantial novel aspects of female germline preservation and development.

## Author Summary

Many aspects of mammalian female germline development during embryogenesis and throughout adulthood are either unknown or under debate. In this study we applied a novel method for the reconstruction of cell lineage trees utilizing microsatellite mutations, accumulated during mouse life, in oocytes and other cells, sampled from young and old mice. Analysis of the reconstructed cell lineage trees shows that oocytes are clustered separately from bone-marrow derived cells, that oocytes from different ovaries share common progenitors, and that oocyte depth (number of cell divisions since the zygote) increases significantly with mouse age.

## Introduction

Understanding the complex processes of embryonic development and post-natal maintenance in multi-cellular organisms requires advanced methods for cell lineage reconstruction. The mammalian female germline is a prominent example, in which fundamental aspects of these processes remain debatable. Unlike lower metazoans such as *C. elegans* and *Drosophila*, in which germ-cell progenitors are set aside during the very first embryonic divisions, in mice, primordial germ cells (PGCs) appear at a much later stage [1], [2]. The late appearance of PGCs, their long-range migration into the gonadal ridges and their co-occurrence with progenitors of hematopoietic and mesenchymal stem cells within the aorta-gonad-mesonephros region of the developing embryo, raised the intriguing possibility that these cell populations may be clonally related [3], [4]; however this hypothesis as well as the modes of expansion and migration of PGCs to the gonadal ridges [5] has thus far not been experimentally tested.

An additional aspect which remains poorly characterized is related to folliculogenesis, the process by which ovarian follicles mature and are selected for ovulation. Folliculogenesis begins with primordial follicles that contain a single layer of squamous pre-granulosa cells that surround an oocyte. Follicles grow through primordial, primary and secondary stages before they develop an antral cavity. The transition from pre-antral to antral follicle occurs only after puberty and is followed by cyclic recruitment of a limited, species-specific number of growing follicles, from which a subset is selected for ovulation [6]. The ‘production-line’ hypothesis suggests that the order by which follicles are selected for growth follows the order at which their oocytes embark on meiosis during embryogenesis, but evidence supporting this notion is sparse [7]–[10]. The ability to reconstruct phylogenies of individual cells and to infer the number of divisions they have undergone since the zygote can address these fundamental questions.

While the de-novo generation of oocytes has traditionally been considered to cease during fetal development in most mammals [11], several recent publications argued for continuous post-natal oocyte renewal in the mouse. These studies were based on several lines of evidence, mainly the discordant proportion between oocyte death and their depletion [12], [13], and the detection of primordial germ cell markers in conjunction with proliferative markers in different cell populations in the ovary [12], [14] and in bone marrow and peripheral blood [15]. These findings were challenged by several publications that failed to reproduce some of the reported observations [16]–[18]. Recently, putative mouse germline stem cells were successfully cultured and transplanted into ovaries of subfertile mice, giving rise to offspring of donor origin [14], suggesting that cells in the adult mouse retain the capacity for oogenesis. However the existence, source and contribution of germline stem cells during normal development remain unclear.

We have previously developed a high-throughput method that uses the information encoded in somatic mutations to reconstruct cell lineage trees [19]–[22]. This phylogenetic method, which was also applied by others [23]–[26], is based on the notion that the DNA is a molecular clock which effectively counts the number of mitotic divisions a cell has undergone since the zygote (denoted as “depth”) and that the pattern of somatic mutations in multiple loci can reveal the lineage relations among individual cells. Our analysis is based on somatic mutations accumulated in microsatellites (MS) loci that reside in intergenic regions. Since these mutations do not affect genes, they are not expected to cause phenotypic effects, and can thus serve as neutral developmental molecular clocks. Our method was validated using ex-vivo cell lineage trees [19] and applied to the lineage analysis of cells of a mouse with a tumor [20], as well as to the estimation of depth of different cell populations [22], and the study of the development of muscle stem cells. Most recently, we demonstrated the reliability of this method for the detection of stem cells and tissue dynamics in the colon [27].

Here we apply this method to address the lineage relations of oocytes and other cell types. We sampled more than 900 cells from 16 mice spanning a range of ages. Sampled cells included oocytes, bone-marrow derived mesenchymal stem cells and lymphocytes, cumulus cells (the epithelial cell surrounding the oocytes in the ovarian follicle) and pancreatic islet cells.

We found that in the reconstructed cell lineage trees of mice at all ages, oocytes form clusters that are distinct from other cell populations. Oocytes from the two ovaries, however, do not form two distinct clusters, suggesting a spatially-incoherent mode of expansion and migration of their embryonic progenitors. In the reconstructed cell lineage trees, the depth of oocytes increases with mouse age and this increase is accelerated in mice that have undergone unilateral ovariectomy. Two alternative explanations can possibly account for the age-associated depth increase, one of which is post-natal oocyte renewal from germline stem cells. The alternative interpretation of our results would go along with a depth-guided oocyte selection, posing that in a sexually mature female mouse oocytes are selected to resume meiosis according to the order in which they embarked on meiosis during embryonic life [7].

## Results

### Oocytes form a cluster distinct from bone marrow cells

In the current application of our method, the cellular genomic signature is derived from a set of MS loci in mismatch-repair (MMR) deficient mice (mlh1−/−). The MS mutation rate of these mice is much higher than that of wild type [28], thus increasing the precision of the cell lineage analysis. These mice are infertile and develop cancer spontaneously, however they display normal ovarian histology [29], [30] (Figure S4). Recently, it was shown that on the background of C3H, the oocytes of mlh−/− mice complete the first meiotic division as indicated by the formation of the first polar body in a fraction which is similar to that of oocytes of a wild type mouse [31]. This is unlike a previous report demonstrating that oocytes of mlh−/− mice on a B6 background fail to resume meiosis [28]. Similarly to mice on the C3H background, oocytes of mice used in this study, which are on a dual background of B6 and M129, complete the first meiotic division, with 100% of the ovulated oocytes extracted from the oviducts displaying a polar body (Table S3).

We sampled oocytes from 17 mice at different age groups ranging from 12 days to one year old (Table S1). These oocytes were arrested in prophase of the first meiotic division containing a nuclear structure known as germinal vesicle (GV). These oocytes were isolated from Graaffian follicles of sexually mature mice, as well as from pre-antral follicles in younger animals. Few ovulated oocytes, arrested at the second metaphase were recovered from the oviduct. In addition, we isolated other cell types, including mesenchymal stem cells, lymphocytes extracted from the spleen, thymus and lymph nodes, and cumulus cells (the inner layer of follicular epithelial cells surrounding the oocyte).

The DNA of all cells was amplified over a panel of 81 microsatellite loci (Table S2) and the size of each allele was determined, thus providing a genomic signature which is the deviation from the putative zygote in the number of microsatellite repeats at each locus. The signatures were used to reconstruct lineage trees using a maximum likelihood Neighbor Joining algorithm (Materials and Methods) and the resulting trees were used to estimate depth (the number of somatic cell divisions since the putative zygote). The genomic signature of the putative zygote was taken as the median of the signatures of all sampled cells. Relative depth was converted to absolute depth (actual number of cell divisions) by calibrating the system on an ex-vivo tree in which the number of divisions is known [22] (Text S1).

**Figure 1:**
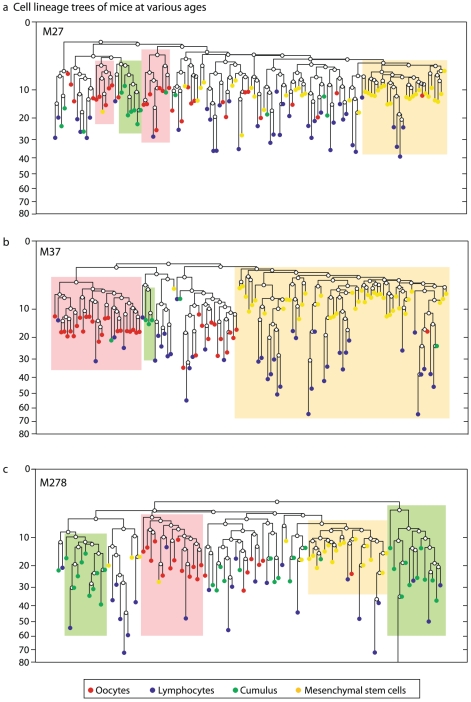
Oocytes form a cluster distinct from bone marrow cells. Reconstructed cell lineage tree of mice in several ages, each showing that GV oocytes from large antral follicles (red) form a cluster that is distinct from cells of bone marrow origin (mesenchymal stem cells- yellow and lymphocytes-blue). Ovarian cumulus cells are in green. Shaded boxes denote subtrees that are statistically enriched for cells of a certain cell population, using a hyper-geometric enrichment test. Mouse names represent their age in days. Y axis is depth (number of divisions since the zygote). One cell in M278 was cropped for visual clarity.

We first examined whether different cell populations form clusters on the lineage tree, by testing whether subtrees are enriched with a given cell population (Materials and Methods). Such a cluster of a cell population would suggest a small number of embryonically distinct progenitors (Text S2, Figures S2, S3). In all reconstructed cell lineage trees of all mice, young and old, oocytes from large antral follicles form a cluster that is distinct from the clusters formed by hematopoietic cells and mesenchymal stem cells (Figure 1).

We found that clustering of cell samples on the reconstructed lineage trees is indicative of the number of progenitors of the cell population studied (Text S2, Figure S1, S2 and S3). The larger the number of progenitors, the less significant clustering observed. The way that subsamples of oocytes form clusters suggests that the number of progenitors of this population is between 3 and 10 (Figure S3), in line with previous estimates based on measurements of primordial germ cells [1], [32]–[35]. Our clustering results indicate that the primordial germ cell lineage is a polyclonal population descendant from a few progenitors, which is embryonically distinct from hematopoietic and mesenchymal stem cells and does not significantly contribute to bone-marrow stem cell populations.

### Oocytes from the two ovaries do not form two distinct clusters

We next turned to examine the clustering of oocytes sampled from different ovaries with the aim of unveiling the dynamics of primordial germ cell expansion and their migration during fetal life.

**Figure 2:**
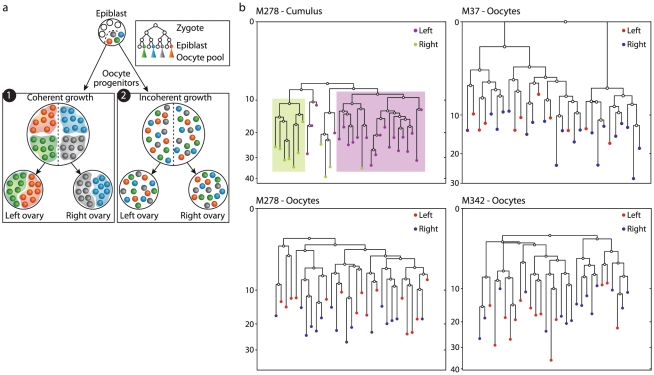
Lack of lineage barriers between oocytes sampled from the left and right ovary suggests a spatially incoherent mode of expansion of primordial germ cells during embryonic development. a) Two modes of expansion-migrations of a small pool of progenitors which become lineage restricted during the epiblast stage. In a spatially coherent mode (1) progenies remain physically close together as they expand in numbers and thus separation into the two ovaries creates two distinct populations. In a spatially incoherent mode (2) progenies physically mix and thus separation into the two ovaries result in equal samples of the original progenitors. b) Reconstructed cell lineage trees show that GV oocytes from large antral follicles of the right (blue) or left (red) ovaries do not form distinct clusters. As a positive control cumulus cells sampled from two follicles – one in the right ovary (green) and one in the left (purple) do form distinct clusters, as expected given the previously estimated small number of progenitors of the epithelial population of a given follicle.

The progenitors of PGCs are set aside at the pre-gastrulation epiblast stage [1], [32] and then undergo rapid expansion while migrating to the gonadal ridges [5], where they separate to the left and right gonads. A cluster of oocytes from one ovary would be indicative of a spatially coherent migration of PGCs, with minimal physical mixing of the progenies of given clones (Figure 2a1). In contrast, if pairs of cells from the same ovary were not closer on the reconstructed lineage trees than mixed pairs, this would suggest a spatially incoherent mode of expansion-migration. Such a mode entails physical mixing of the progenies of dividing PGCs as they migrate before their allocation to different gonads. In this scenario the populations of both ovaries would form an identical sampling of the progenies of the different founder clones (Figure 2a2). As a positive control we reconstructed cell lineage trees of cumulus cells extracted from two different follicles, one from each ovary. The population of cumulus cells in a given follicle has been shown to originate from only five progenitors [36] and is thus expected to form a cluster on a reconstructed lineage tree.

Our analysis of the reconstructed cell lineage trees revealed that while cumulus cells sampled from different follicles form distinct clusters (Figure 2b), oocytes from large antral follicles from the left and right ovaries never do (Figure 2b, Figure S5). This result suggests a mode of incoherent clonal expansion during the process of primordial germ cell migration to the gonadal ridges, as depicted in Figure 2a2.

While primordial germ cells undergo several additional rounds of mitotic divisions after having settled in the gonadal ridges thus giving rise to small clones, the lack of lineage clustering between the two ovaries suggests that at this stage the number of such clones is significantly larger than the number of oocyte sampled here. Thus the probability to sample more than one oocyte from a single clone is small (Figure S2).

### Oocyte depth increases with mouse age

**Figure 3:**
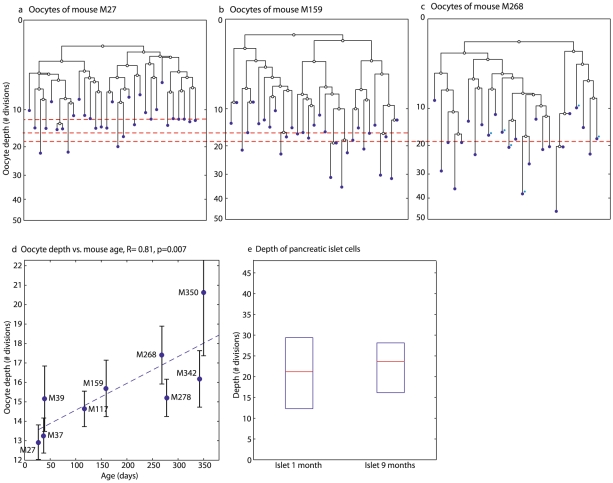
Oocyte depth increases with age. a–c) Reconstructed lineage trees of GV oocytes from large antral follilces in three mice demonstrate an increase in oocyte depth (Y axis) with age. The putative zygote is at depth 0. Median depth of oocytes is 13 divisions in M27, a 27 day old mouse (Mouse names represent their age in days) (a), 16 divisions in mouse M159 (b) and 19 in mouse M268 which contains 6 ovulated oocytes which were marked on the tree with blue dots (c). Horizontal red lines denote the median depth. All lineage trees were reconstructed using the maximum-likelihood neighbor joining method and rooted with the median identifier of all cells. d) Median depth of GV oocytes from large antral follicels increases with age. Each blue point is the median of the depths of all oocytes sampled from a single mouse. The Pearson correlation of median depth and age is 0.81 (p = 0.007). High inter-mouse variability can be seen. Errorbars are standard errors of the means. (e) Depth of pancreatic islet cells does not increase with age. Shown are the depths of pancreatic islet cells from M36, a 1-month old mouse and from M280, a 9-month old mouse.

The topology of reconstructed lineage trees can shed light on the developmental processes of the oocyte lineage. To address the possibility of post-natal oocyte renewal during adulthood we next analyzed the depth of oocytes sampled from large antral follicles of mice at different ages. Analysis of the reconstructed cell lineage trees showed that oocyte depth increases significantly with mouse age (Figure 3, R = 0.81, p = 0.007, bootstrap R = 0.56).

Unlike oocytes, the depth of pancreatic islet cells, which have been shown to have a low turnover rate in adult mice [37], [38], does not increase with age (Figure 3e). On the other hand, epithelial intestinal cells display a substantial increase in depth from age 1 to 11 months [27]. The depth of other cell types sampled also increases during adulthood (Figure S6).

**Figure 4:**
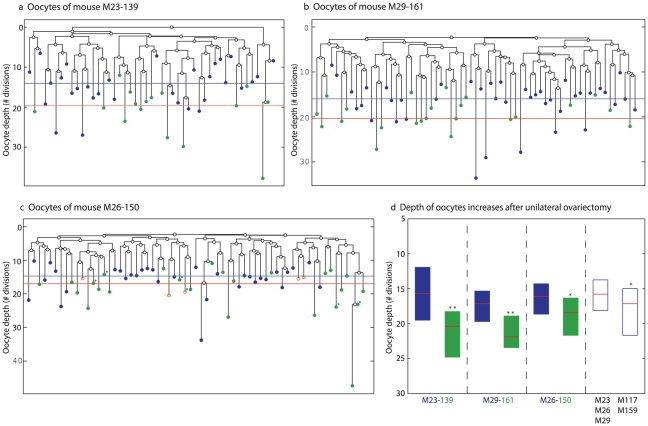
Accelerated increase in GV oocyte from large antral follicles depth following unilateral ovariectomy. a–c) Reconstructed lineage trees of oocytes from both ovaries of unilaterally ovariectomized mice. Blue circles are oocytes extracted from the ovary removed at a young age – 23 days (a) 29 days (b) and 26 days (c). Green circles are oocytes extracted from the contralateral ovary removed at an older age – 139 days (a) 161 days (b) and 150 days in which the ovulated oocytes were marked with blue dots (c). Horizontal lines are the average depths of young oocytes (blue) and old oocytes (green). d) Increase in depth with age following ovariectomy is accelerated relative to the increase observed for non-ovariectomized mice. Last two boxes include pooled data from the indicated mice. * p<0.05, ** p<0.01.

To control for the apparent inter-mouse depth variability (Figure 3d), we performed three longitudinal unilateral ovariectomy experiments in which one ovary was removed when the mouse was one month old, whereas the second ovary of this same mouse was removed at the age of four months. Analysis of the reconstructed cell lineage trees revealed that oocytes from large antral follicles harvested from the ‘old’ ovary are significantly deeper than such oocytes harvested from the ‘young’ ovary (Figure 4). Interestingly, the depth of oocytes from the ‘old’ ovary is significantly higher as compared to oocyte depth in mice at similar age that did not undergo this intervention (Figure 4d).

#### Increase in oocyte depth is not due to spontaneous mutations

We next considered the possibility that the observed increase in microsatellite mutations in older oocytes is due to spontaneous mutations that is not dependent on cell division [39], [40]. To validate that our microsatellite loci do not accumulate mutations in cells with a low rate of replication, we analyzed pancreatic islet cells, which were previously shown to have low turnover in adult mice [37], [38]. We found that pancreatic islet cells do not accumulate microsatellite mutations with age (Figure 3e).

**Figure 5:**
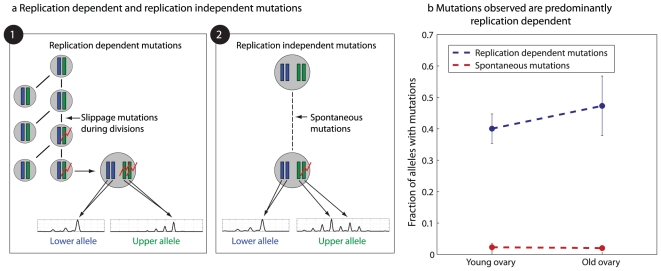
Microsatellite mutations are replication dependent. a) Shown are two scenarios for microsatellite mutations – replication dependent mutations that occur during mitotic divisions at a cell stage with two chromosomal copies (left), and spontaneous, replication independent mutations occurring at the quiescent oocyte stage with four chromosomal copies (right). While replication dependent mutations would result in two alleles, spontaneous mutations would mostly result in more than two alleles. The bottom plots show representative capillary signals. b) The increase with age in fraction of alleles with spontaneous mutations is significantly smaller than the increase in fraction of alleles with replication dependent mutations. Results are for the three longitudinal experiments.

Further analysis was done to rule out the possibility that oocytes may accumulate spontaneous mutations at a higher rate than other somatic cells. The ability to detect spontaneous mutations in oocytes is facilitated by the fact that meiotically arrested, GV oocytes contain four copies of each locus rather than two (two copies for each of the two parental alleles). A spontaneous mutation that occurs during the prolonged phase of meiotic arrest would, most probably, hit only one chromosome of the four, resulting in a locus with more than two alleles. Therefore if most loci have only two alleles one can deduce that mutations have occurred in an oocyte progenitor, a stage with only two copies per locus (Figure 5a).

The analysis shows that the number of loci in which more than two alleles were observed (representing spontaneous mutations at the four allele stage) is an order of magnitude less than the total number of observed mutations (Figure 5b, Figure S7), suggesting that their role in the observed depth increase is negligible. Moreover, since all cases in which more than two alleles per locus were observed, were eliminated from our analysis, the observed increase in depth predominantly represents mutations that accumulate with cell divisions at a mitotically active progenitor, containing two alleles per locus.

The fact that oocytes in unilaterally ovariectomized mice accumulate mutations faster than in untreated mice is another indication that the mutations accumulated are not spontaneous, which would accumulate at the same rate following this treatment. In addition, we observe no increase in mutations with age in wild-type mice (Figure S8), ruling out the possibility that the mutations we observe are spontaneous and independent of the mlh1-defficiency-related mutation in our mice. Finally, the pattern of mutations observed in all experiments is consistent with a symmetric step-wise model, as expected from microsatellite mutations coupled with DNA replication [41] (Figure S9).

### Depth-guided maturation versus post-natal renewal

By rejecting the explanation of spontaneous mutations for the increase with age in accumulated somatic mutations, we conclude that oocytes in older mice are deeper (undergo more mitotic divisions) than oocytes in young mice. Such divisions can occur at either embryonic development or during adult life. We next consider these two possibilities.

**Figure 6:**
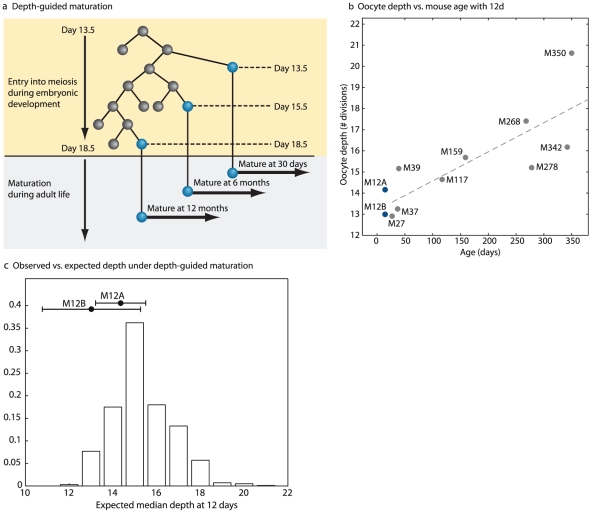
Depth-guided oocyte selection for ovulation accounts for the increase in oocyte depth with age. (a) Depth-guided oocyte maturation. Shown is a hypothetical tree showing entry into meiosis during embryonic development. The hypothesis states that oocytes that enter meiosis first during embryonic life would be ovulated first during post-natal life. (b) Comparison of median depth of oocytes isolated from pre antral follicles isolated from 12 day-old mice vs. oocytes isolated pre ovulatory follicles isolated from older mice. Each point is the median of the depths of all oocytes sampled from a single mouse. (c) Depth distributions of oocytes from pre-antral follicles in 12 day-old mice are not significantly different from the expectation from the production line hypothesis. Shown is the expected distribution of median depth of oocytes assuming that the depth distribution at birth is a uniform average of all measured post-natal depth values of maturing oocytes. Filled circles denote the observed median depth in the two 12 day old mice. Error bars are standard errors of the means.

The ‘production-line’ hypothesis introduced by Henderson and Edwards [7], [10], [42], posits that the order at which oocytes are ovulated during adult life follows the order at which PGCs enter meiosis during fetal life. This would result in a lower depth of oocytes from large antral follicles in young mice as compared to oocytes recovered from such follicles of old mice (Figure 6a). Indeed, both, the order at which germ cells enter meiosis and the growth of primordial follicles have been suggested to be spatially structured [43], [44] and thus not completely arbitrary. However, the exact sequence and spatial details of these two processes have not been firmly resolved [42]. While the oocytes sampled in this study were taken from large antral follicles selected for ovulation, a wide distribution of oocyte depth extracted from pre-antral follicles at birth would be suggestive of depth-guided oocyte maturation.

To examine this possibility we sampled oocytes from 12 day-old mice, an age at which the entire population of the ovarian follicles is at the pre-antral stage. Thus, our sampling in these mice was not enriched for follicles selected for ovulation. We found that the median depth of two 12 day-old mice was similar to the median depth of young animals (Figure 6b, Figure S15). To compare the depth distributions in pre-antral follicle oocytes in 12 day old mice to that expected from the ‘production-line’ hypothesis we performed the following simulation – a putative depth distribution was create based on a uniform weighting of the depth values of oocytes measured at different ages. The depth of values sampled from this distribution was compared to that seen in the 12 day old mice, and the fraction of simulated values for which the maximal/median depth was smaller was reported as a p-value. We used Fisher's method for combining the p-values of two 12-day old mice. We found that the distribution of oocyte depths of pre-antral follicles from 12 day old mice is not statistically different from that expected from a pre-existing wide distribution that spans the entire depth range seen for all ages (Figure 6c). As such our results do not conclusively rule out the ‘production-line’ hypothesis indicating that depth-guided maturation could give rise to the observed increase in oocyte depth with age [12], [13].

## Discussion

The ability of the germline to generate all somatic cell types, the analogous ability of bone-marrow derived stem cells to differentiate to a wide range of cells [45], [46], and the physical proximity of the progenitors of these populations during embryonic development, raised the intriguing possibility that these different cell populations are the progenies of a common precursor [3], [4]. Here, using cell-lineage trees reconstructed from somatic mutations, we show that the mouse female germline is embryonically distinct from cells derived from bone marrow stem cells, be they mesenchymal or hematopoietic stem cells. Thus, this study indicates that progenies of primordial germ cells do not contribute to bone-marrow stem cell populations. In addition, this study suggests that putative germline stem cells that may contribute to post-natal oocyte renewal are progenies of the primordial germline, rather than being of bone marrow origin [15]. We considered analyzing cells from the ovarian surface epithelium, which were suggested to harbor the germline stem cells. However because germline stem cells are expected to constitute a small fraction of this cell compartment [47] and thus our chances of sampling them are very low, and since we must analyze each mouse separately to obtain an informative lineage tree, we could not pursue this direction in our system. Similar cell-lineage analysis of other cell populations, such as that presented here could shed light on the embryonic development and post-natal dynamics of other tissues and cell populations, providing novel insight into the architecture of multi-cellular organisms.

The reconstructed cell lineage trees revealed that oocytes from older mice undergo more mitotic divisions since the zygote as compared to oocytes of young mice. This finding may point towards post-natal oocyte renewal. However, since sampling in our study was directed at oocytes that reside in the large antral follicles, selected for ovulation, the age-associated increase in their depth could also represent depth-guided oocyte selection [7]. The depth distribution of oocytes recovered from from pre-antral follicles of 12-day-old mice does not deny the possible depth-guided oocyte selection, thus leaving the two optional interpretations of our results open. Further cell lineage analysis studies, for example using a mouse model susceptible to post-natal induction of MMR-deficiency, are needed to decide between the two.

While previous evidence regarding the ‘production-line’ hypothesis linked the time of entry into meiosis with the time of post-natal oocyte maturation [9], [10], [42], the present study suggests that under the hypothesis of depth-guided oocyte maturation, the number of divisions rather than the embryonic day, determines the order of oocyte maturation during adult life. Similar cell-lineage analysis of other cell populations, such as that presented here, could shed light on the embryonic development and post-natal dynamics of other tissues and cell populations, providing novel insight into the architecture of multi-cellular organisms.

The accelerated increase in oocyte depth in the unilaterally ovariectomized mice may be related to the previously described phenomenon of doubling the rate of ovulation from the contralateral remaining ovary that is a subsequent to a systemic elevation of pituitary stimulating gonadotropins [48]. Our study implies that under these conditions the remaining ovary is populated by deeper oocytes. Along this line, it has been shown that hormonally-stimulated super ovulation results in oxidative damage to DNA and mitochondrial DNA mutations [49]. Thus, exposure to high doses of stimulating hormones, as is often practiced to treat infertility, may severely impair oocyte quality by introducing into the oocyte pool deeper oocytes.

In summary, we present a comprehensive analysis of the mouse oocyte lineage at the single cell level, addressing open questions regarding both the development and post-natal maintenance of female gametes. Our analysis revealed that oocytes are clustered distinctly from bone-marrow derived cells, that progenitors of oocytes from different ovaries are mixed, that oocyte depth increases significantly with mouse age, and that this increase is accelerated after ovariectomy. Our methodology can be used to infer the early developmental processes and post-natal clonal dynamics of other tissues and cell populations.

## Materials and Methods

### Animals and ethics

C57Bl/6 mice, Mlh1+/− (kind donation of Prof. Michael Liskay) [50] and 129SvEv mice, Mlh1+/− (kindly provided by Prof. Ari Elson from the Weizmann Institute, Israel) were mated to yield Mlh1−/− progeny of the dual backgrounds, enabling us to distinguish, in all our experiments, between two alleles in the same locus. All animal husbandry and euthanasia procedures were performed in accordance with the Institutional Animal Care and Use Committee at the Weizmann Institute of Science.

### Isolation and analysis of single cells

Mice were not superovulated in order to avoid any effect of external hormones on our analysis. Ovaries were removed and placed in Leibovitz's L-15 tissue culture medium (Gibco), supplemented with 5% fetal bovine serum (Biolab, Jerusalem, Israel), penicillin (100 IU/ml) and streptomycin (100 µg/ml, Gibco). The follicles were punctured under a stereoscopic microscope in order to release the cumulus–oocyte complexes that were then placed into acidic L-15 medium (pH 6.0) to obtain cumulus-free oocytes (Figure S14). Most of the oocytes were MI-arrested from pre-antral and from develpme, thus retaining all four meiotic products. In few cases ovulated oocytes were isolated from the oviduct and their polar body was seen and it was extracted together with the oocyte. Our oocyte samples in M268 and M26–150 included a few ovulated oocytes that have extruded their polar body (blue dots, Figure 3c and Figure 4b). Excluding them had no effect on median oocyte depths in these mice. Each oocyte was placed in a 0.2 mL tube (ABgene) in a volume of 2 µL medium. Oocytes were frozen in liquid nitrogen and kept in −80°C until DNA ampification. In the longitudinal experiment, mice were anesthetized and then underwent unilateral ovariectomy. The section was clipped and after additional 4 months the other ovary was removed when the animal was sacrificed.

Mesenchymal stem cells, B-cells, NK-cells and T-cells were isolated as described in [21]. Pancreatic islet cells where extracted from 1-month old and 9-month old male mice using single cell laser capture microdissection of Hematoxylin stained tissue, as described in [21].

DNA extracted from individual cells was amplified using whole-genome amplification (WGA) with the GenomiPhi DNA amplification kit (GE Healthcare, UK) as described in [23]. Aliquots of WGA products were used directly (without purification) as templates in subsequent PCRs. PCR repeats and negative controls (DDW) were included in every PCR plate (Figure S10 and Text S3 and Text S4). Loci that exhibited a signal in the negative control were excluded from the analysis of all samples that were run on the corresponding PCR plate. The method achieved high-throughput through the use of a liquid handling robotic system.

### Tree and depth reconstruction

Microsatellite loci were chosen to have a substantial allele size difference between the two mice strains, so as not to confound allele sizing (see Figure S8 for representative capillary signals). Whole genome amplification introduced very few microsatellite mutations, but resulted in allelic drop-out of 32.5+−5%. Capillary signals that displayed more than two alleles per locus were excluded from the analysis. In addition only cells in which more than 25 alleles were amplified were included in the analysis (998 cells, Table S1). Trees were reconstructed using the distance-based neighbor joining algorithm [51], [52]. Pairs of cells were sequentially merged according to a distance matrix of lineage distances. Each entry in the distance matrix is taken as the maximum likelihood estimate of the number of divisions separating the two cells, assuming a symmetric stepwise model and an average mutation rate estimated from the ex-vivo trees, equal to 1/30 divisions (Text S1). Depth was read off the trees as the branch lengths leading from the root to each terminal leaf. Root signature was taken as the median of the allele size values of all sampled cells. This method was found to be more efficient and robust than estimating depth using a squared distance metric (Text S5).

It is important to note that we estimated the microsatellite mutation rate in this study to be one mutation per 30 cell divisions; this estimation is based on our measurements of cell divisions in ex-vivo trees (Text S1). Nevertheless, to be sure that our conclusions are not dependent on this specific mutation rate, we reconstructed trees using different mutation rates ranging from 1 mutation per 10 cell divisions to 1 mutation per 200 cell divisions. Our conclusions (i.e. the depth increase with age, the enrichment of oocytes on the lineage tree and the mixing of oocytes between left and right ovary) are robust to different mutation rates (Figure S11, S12, and S13).

While we have previously estimated a higher oocyte depth [22] our current study used a dramatically increased resolution for depth estimation, based on a larger and more informative panel of high-mutation rate microsatellite loci as well as a larger oocyte sample size.

### Statistical analysis

P-value for the Pearson correlation between median depth and age was based on permutations of the median depth values. Bootstrap correlation coefficient of depth vs. age was obtained by generating 1000 median depth values extracted from sampling with replacement of the cell depths for each mouse. P-values for differences in distributions were calculated using Kolmogorov-Smirnov method. Hypergeometric tests were carried out for each internal branch to assess whether subtree leafs are enriched for a cell population. P-values declared as significant were corrected for multiple hypothesis testing using false discovery rate of 0.2. Whenever subtrees were embedded only the subtree with the most significant p-value was retained. Noteworthy, clustering of oocytes is stronger in M37 and M278 relatively to M27. We examined whether this could be attributed to allelic dropout, however we did not find any significant difference between the average amplified loci per cell in these different mice (42+−5, 51+−9 and 43+−5 in M27, M37 and M278, respectively). The variability in lineage clustering is strongly dependent on the number of cells sampled from each population and on the number of progenitors. Since oocytes are a polyclonal population, sampling in different mice could give rise to either more cells from fewer clones (leading to strong lineage clustering, Figure S2 and S3) or alternatively cells distributed equally among the progenitor clones (leading to weaker clustering). In addition, the progenitor clones themselves could be distributed differently on the progenitor lineage tree, thus affecting variability of clustering between animals. This issue is further discussed in Text S5 and Figures S2 and S3.

## Supporting Information

Figure S1 Comparison of depth estimator methods. The estimator bias (a,b) efficiency (c,d) and robustness to deviations from the mutation model (e) and to signal errors (f) is shown. Results are for 30 cells sampled from a binary tree of depth Td = 25 (a,c,e,f) and Td = 45 (b,d), with errorbars over 50 sampling iterations. Estimator variability was measured using the standard error of the mean (SEM) of the depth estimate.
(TIF)

Figure S2 The number of progenitors of a cell population is smaller than the number of sampled cells that reveals lineage clustering. Binary trees of depth 25 divisions were simulated. The figure shows the induced tree on 30 cells randomly sampled from a population that differentiated from Np progenitors that became lineage restricted at the 8^th^ division (red circles), as well as 30 cells randomly sampled from the remaining tree (empty squares). Green branches denote subtrees that are statistically enriched for the red cells (using hypergeometric test and FDR of 0.05). The ability to detect significant lineage clustering decreases as the number of progenitors approaches the number of samples.
(TIF)

Figure S3 Estimating the number of progenitors of oocytes based on lineage clustering. Shown are simulations (solid lines) and real data (dashed lines). For each progenitor value (Np) 50 lineage trees were generated in which Np progenitors were randomly selected from the 256 cells at the T = 8 division. For each tree *s* samples from the population and *s* from its complement were randomly chosen, microsatellite mutations were added and the tree reconstructed using the Tree-ML method. Shown are the fraction of trees in which lineage clustering was observed (using hypergeometric tests for each internal branch and an FDR of 0.05). Errorbars are over the 50 sampling iteration. For the real data (mice M27, M37, M278) *s* cells were sampled from the oocyte population and from the rest of the cells. Plotted are the fractions of sampling iterations in which the oocytes are clustered on the reconstructed lineage tree. Error bars are over 30 iterations of this sampling process. Our simulations indicate that the number of oocyte progenitors is within the range of 3–10 progenitors.
(TIF)

Figure S4 Histological section of an ovary taken from a 50 day old mlh1−/− mouse. Follicles at different stages of maturation can be observed.
(TIF)

Figure S5 Oocytes from the right ovary (blue) and left ovary (red) do not cluster on the reconstructed lineage trees.
(TIF)

Figure S6 Depth versus age for different cell types. Median depth shown in red, boxes delimits 25–75 percentiles. Mouse name includes age in days, e.g. M27 is a 27 day old mouse.
(TIF)

Figure S7 Representative capillary signals of four loci for mouse M29–161. Shown are signals for six representative oocytes for each locus. X axis denotes fragment size in base-pairs, y axis denotes capillary signal height (arbitrary units). Cyan and magenta vertical lines denote the size of the lower and upper alleles respectively, determined as the position of the highest peak within the PCR stutter pattern of each allele. Gray vertical line denotes the database expected fragment length. a) ADR-6 (53 repeats of T). b) mX188_GT29 (29 repeats of GT). c) ADR-38 (42 repeats of AG). d) mX138_AG32 (32 repeats of AG).
(TIF)

Figure S8 Lack of increase in oocyte depth in wild-type mice. Shown are the distributions of average square deviation in alleles sizes from the root for a 12 day old mouse (M12D) and a 377 day old mouse (M377). The distributions are not statistically different (pval = 0.3). The amount of mutations is 5-times smaller than that obtained in WT mice (this includes PCR caused mutations, the in-vivo mutation rate is even smaller).
(TIF)

Figure S9 Allele size distributions of a 12 day old mouse (M12B) and a 342 day old mouse (M342). Shown are the fractions of alleles that display different allele size deviation from the signature of the root (putative zygote).
(TIF)

Figure S10 PCR repeats are clustered on the reconstructed lineage tree – each PCR plate included a pair of repeats, with the aim of controlling for the high microsatellite mutation rates that can result in mutations occurring even during the PCR process. The reconstructed lineage tree of M23–139 including PCR repeats (red) demonstrates that PCR repeats are always strongly clustered. Similar results are seen in all trees.
(TIF)

Figure S11 Oocyte depth increases with age using different mutation rates. Shown are five subplots, similarly to Figure 3d. The median oocyte depth of each mouse in each subplot is represented by blue circle was measured by different mutation rate, ranging from 1 mutation per 10 cell divisions to 1 mutation per 200 cell divisions.
(TIF)

Figure S12 Accelerated increase in oocyte depth following unilateral ovariectomy using different mutation rates. Shown are five subplots, similarly to Figure 4d, each subplot contains 4 columns: A. M23–139 B. M29–161 C. M26–150 D. Left - M23, M26, M29. Right - M117, M159. The median of oocyte depth in each animal at each age in each subplot was measured by different mutation rate, ranging from 1 mutation per 10 cell divisions to 1 mutation per 200 cell divisions.
(TIF)

Figure S13 Oocytes form a cluster distinct from bone marrow cells using different mutation rates. Here we demonstrate the clustering results showed in Figure 1, are robust to variations in the mutation rate. In Figure 1 we used mutation rate of 1 mutation per 30 cell divisions in each locus. Shown is representative cell-lineage trees of M37 for a few other mutation rates in the range [1/10–1/200].It can be seen that for all the different mutation rates, oocyte are clustered separately from bone marrow cells. Oocytes are presented in red, mesenchymal stem cells in yellow, lymphocytes in blue and ovarian cumulus cells in green. Shaded boxes denote subtrees that are statistically enriched for cells of a certain cell population, using the same hyper-geometric enrichment test as we used in the main text. Bold branches represent statistical enriched clades on the lineage tree. The most significant p value is indicated. The width of the branches represents clustering significance (wider = lower p value). The Y axis represents depth in logarithmic scale.
(TIF)

Figure S14 Extracted oocytes are free of granulosa contaminations. Shown are amplified PCR products of PDE3A (250 bp) in oocytes, VEGFA (100 bp) in oocytes and in granulosa cells and negative controls for both. Phosphodiesterase 3A (PDE3A), a gene highly expressed in oocytes, was used as a positive control for the quality of oocyte cDNA, and showed marked expression in this sample.
(TIF)

Figure S15 Reconstructed lineage trees of GV oocytes in three mice demonstrate oocyte depth of 12 d mouse relatively to oocyte depth in other ages (Y axis). The putative zygote is at depth 0. Median depth of oocytes is 13 divisions in M27 (a), a 27 day old mouse (Mouse names represent their age in days), 14.7 divisions in mouse M12A (b) and 19.5 in mouse M268 (c). Horizontal red lines denote the median depth. All lineage trees were reconstructed using the maximum-likelihood neighbor joining method and rooted with the median identifier of all cells.
(TIF)

Table S1 Number and types of cells sampled from different mice. * denote wild-type mice.
(DOC)

Table S2 Microsatellite loci used for lineage analysis. ^*^ denote loci that were used in the Whole genome amplification control experiment.
(DOC)

Table S3 All oocytes extracted from the oviducts in the two cycling mice show polar bodies.
(DOC)

Text S1 Ex-vivo calibration experiment.
(DOCX)

Text S2 Lineage clustering provides limits on the number of progenitors of a sampled cell population.
(DOCX)

Text S3 Experimental controls.
(DOCX)

Text S4 Contamination control experiment.
(DOCX)

Text S5 Comparison of different depth estimation methods.
(DOC)
