# Supplementary figures and images for: Cell Lineage Analysis of the Mammalian Female Germline
Source: PLoS Genet. 2012 Feb 23;8(2):e1002477. doi: 10.1371/journal.pgen.1002477 (PMC3285577; doi:10.1371/journal.pgen.1002477)

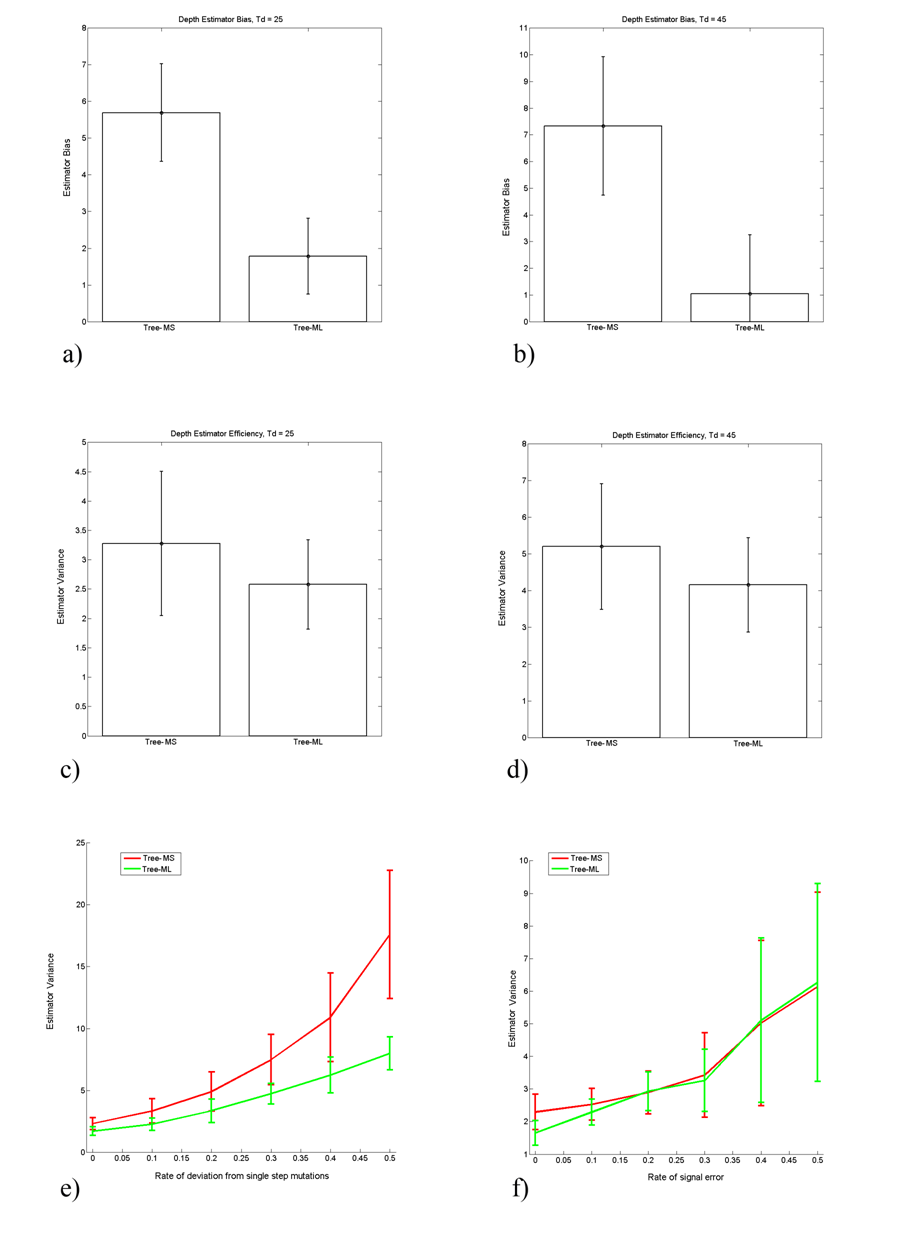

Supplement: Figure S1 — Comparison of depth estimator methods. The estimator bias (a,b) efficiency (c,d) and robustness to deviations from the mutation model (e) and to signal errors (f) is shown. Results are for 30 cells sampled from a binary tree of depth Td = 25 (a,c,e,f) and Td = 45 (b,d), with errorbars over 50 sampling iterations. Estimator variability was measured using the standard error of the mean (SEM) of the depth estimate. (TIF) [file pgen.1002477.s001.tif]

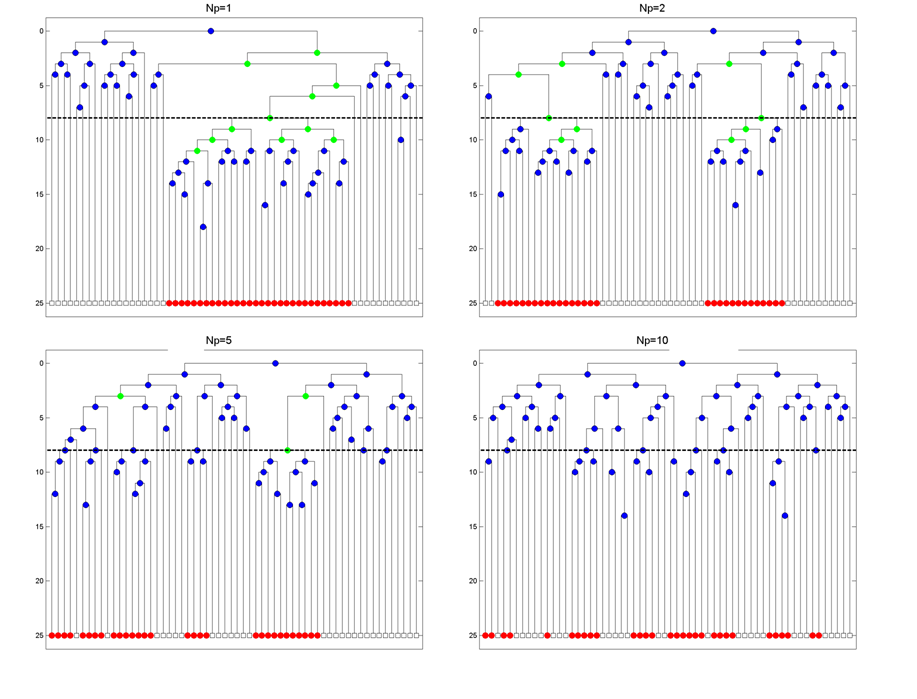

Supplement: Figure S2 — The number of progenitors of a cell population is smaller than the number of sampled cells that reveals lineage clustering. Binary trees of depth 25 divisions were simulated. The figure shows the induced tree on 30 cells randomly sampled from a population that differentiated from Np progenitors that became lineage restricted at the 8th division (red circles), as well as 30 cells randomly sampled from the remaining tree (empty squares). Green branches denote subtrees that are statistically enriched for the red cells (using hypergeometric test and FDR of 0.05). The ability to detect significant lineage clustering decreases as the number of progenitors approaches the number of samples. (TIF) [file pgen.1002477.s002.tif]

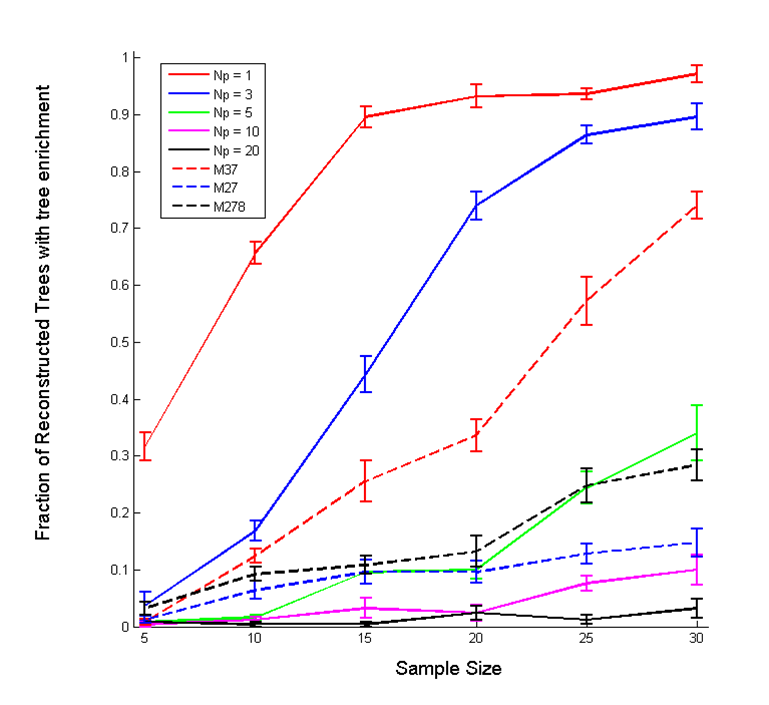

Supplement: Figure S3 — Estimating the number of progenitors of oocytes based on lineage clustering. Shown are simulations (solid lines) and real data (dashed lines). For each progenitor value (Np) 50 lineage trees were generated in which Np progenitors were randomly selected from the 256 cells at the T = 8 division. For each tree s samples from the population and s from its complement were randomly chosen, microsatellite mutations were added and the tree reconstructed using the Tree-ML method. Shown are the fraction of trees in which lineage clustering was observed (using hypergeometric tests for each internal branch and an FDR of 0.05). Errorbars are over the 50 sampling iteration. For the real data (mice M27, M37, M278) s cells were sampled from the oocyte population and from the rest of the cells. Plotted are the fractions of sampling iterations in which the oocytes are clustered on the reconstructed lineage tree. Error bars are over 30 iterations of this sampling process. Our simulations indicate that the number of oocyte progenitors is within the range of 3–10 progenitors. (TIF) [file pgen.1002477.s003.tif]

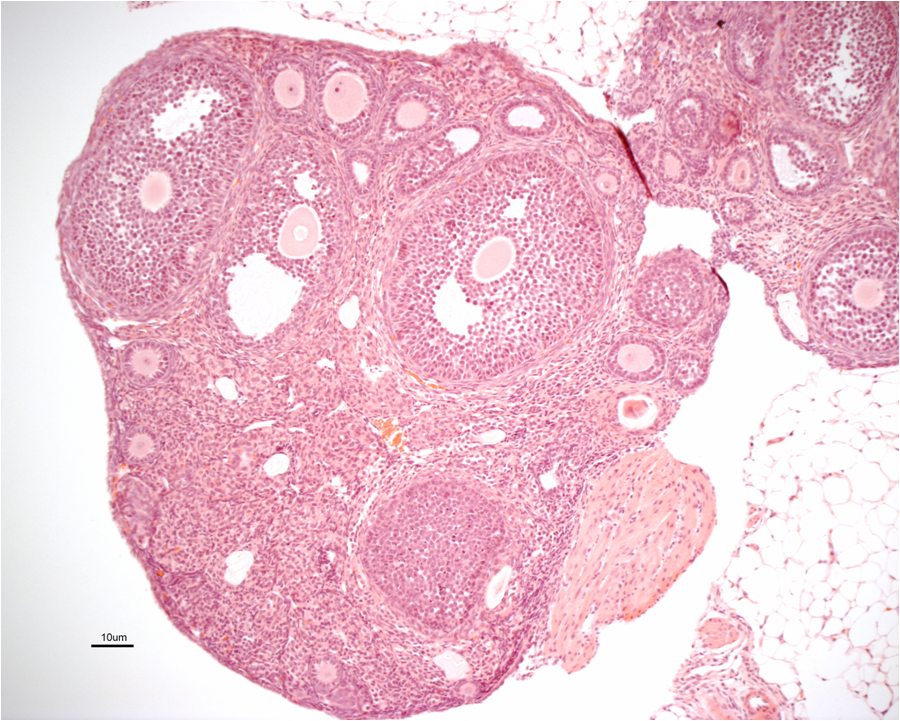

Supplement: Figure S4 — Histological section of an ovary taken from a 50 day old mlh1−/− mouse. Follicles at different stages of maturation can be observed. (TIF) [file pgen.1002477.s004.tif]

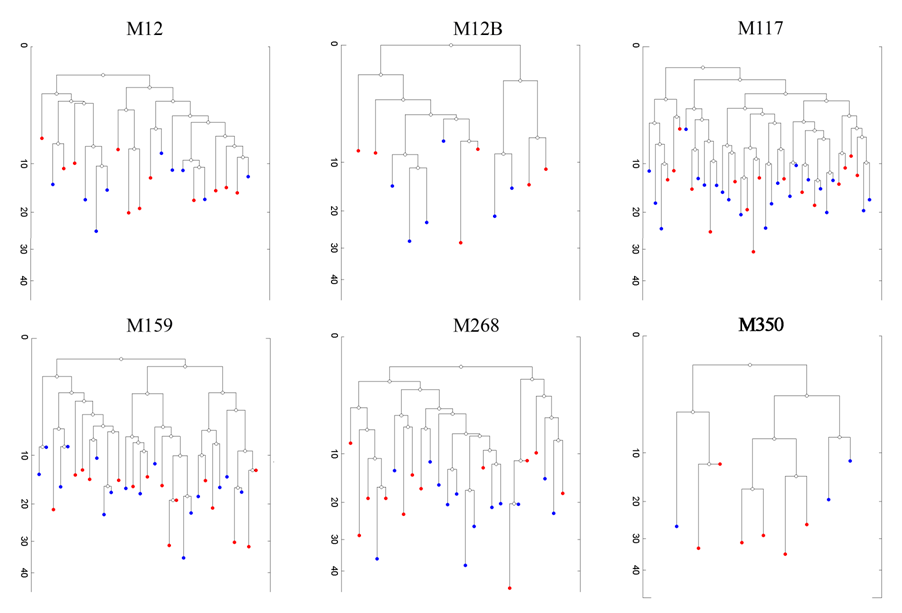

Supplement: Figure S5 — Oocytes from the right ovary (blue) and left ovary (red) do not cluster on the reconstructed lineage trees. (TIF) [file pgen.1002477.s005.tif]

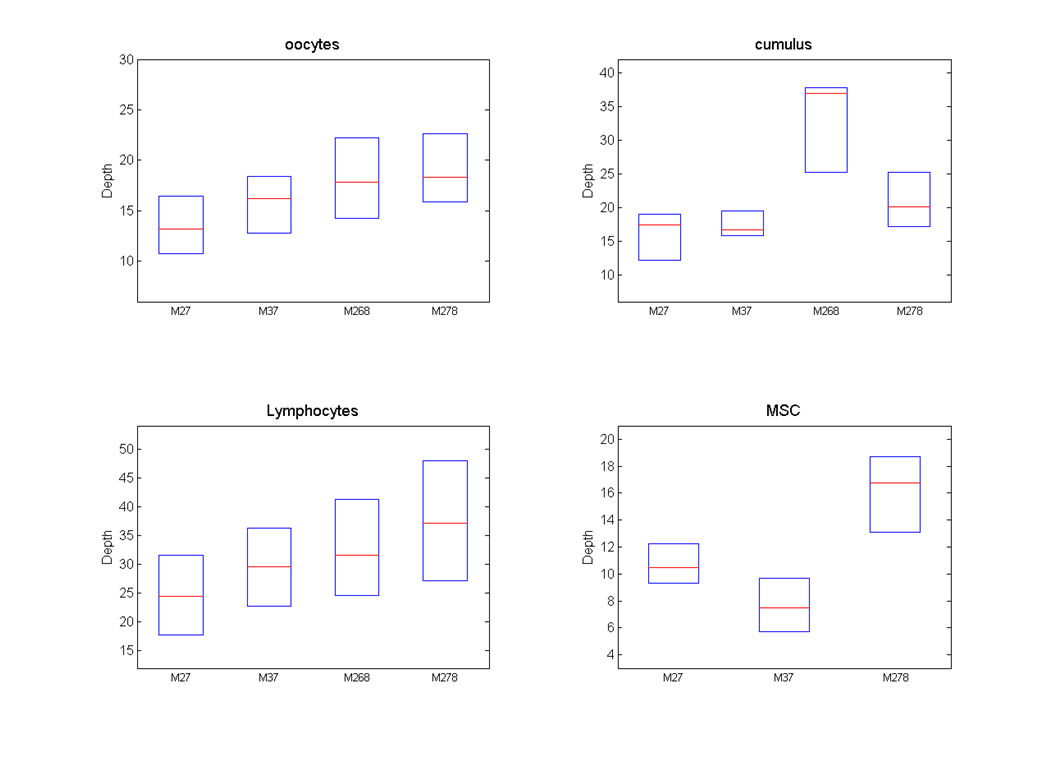

Supplement: Figure S6 — Depth versus age for different cell types. Median depth shown in red, boxes delimits 25–75 percentiles. Mouse name includes age in days, e.g. M27 is a 27 day old mouse. (TIF) [file pgen.1002477.s006.tif]

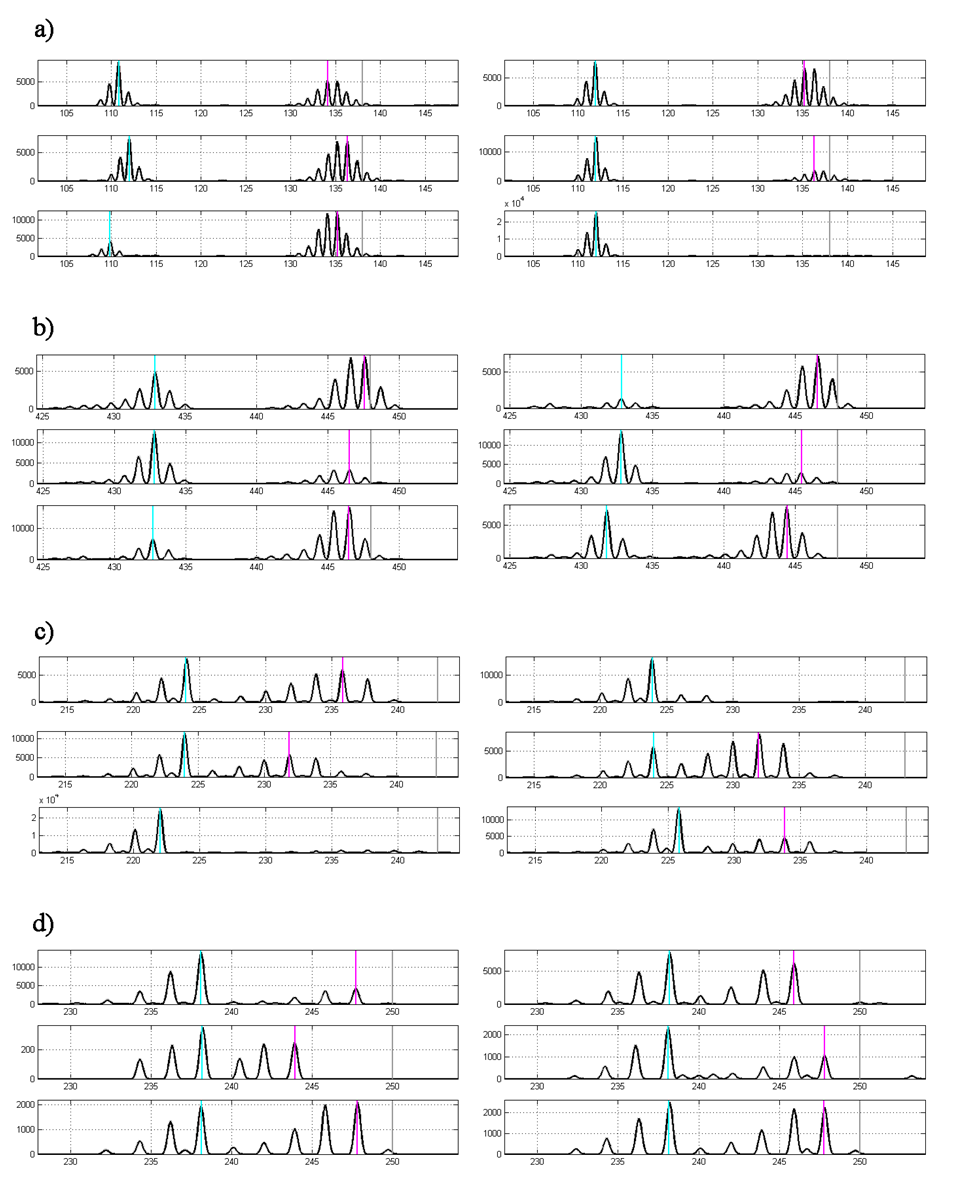

Supplement: Figure S7 — Representative capillary signals of four loci for mouse M29–161. Shown are signals for six representative oocytes for each locus. X axis denotes fragment size in base-pairs, y axis denotes capillary signal height (arbitrary units). Cyan and magenta vertical lines denote the size of the lower and upper alleles respectively, determined as the position of the highest peak within the PCR stutter pattern of each allele. Gray vertical line denotes the database expected fragment length. a) ADR-6 (53 repeats of T). b) mX188_GT29 (29 repeats of GT). c) ADR-38 (42 repeats of AG). d) mX138_AG32 (32 repeats of AG). (TIF) [file pgen.1002477.s007.tif]

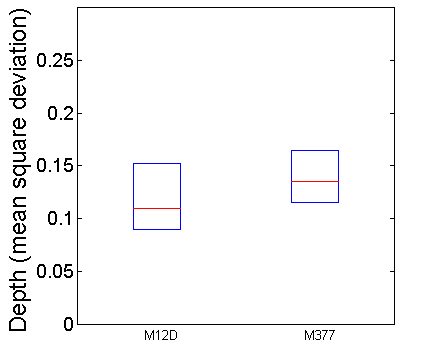

Supplement: Figure S8 — Lack of increase in oocyte depth in wild-type mice. Shown are the distributions of average square deviation in alleles sizes from the root for a 12 day old mouse (M12D) and a 377 day old mouse (M377). The distributions are not statistically different (pval = 0.3). The amount of mutations is 5-times smaller than that obtained in WT mice (this includes PCR caused mutations, the in-vivo mutation rate is even smaller). (TIF) [file pgen.1002477.s008.tif]

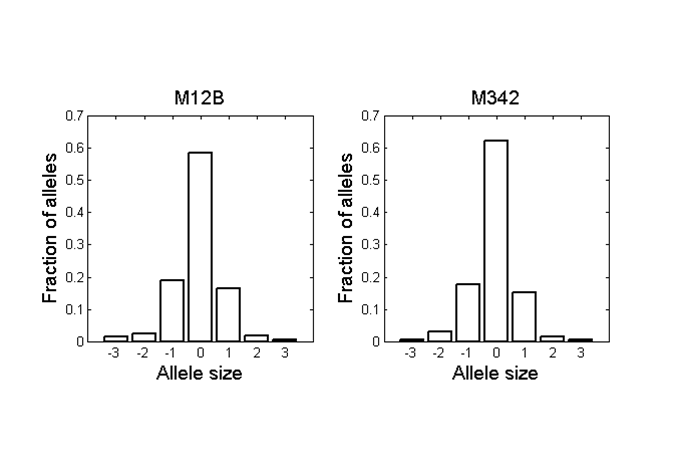

Supplement: Figure S9 — Allele size distributions of a 12 day old mouse (M12B) and a 342 day old mouse (M342). Shown are the fractions of alleles that display different allele size deviation from the signature of the root (putative zygote). (TIF) [file pgen.1002477.s009.tif]

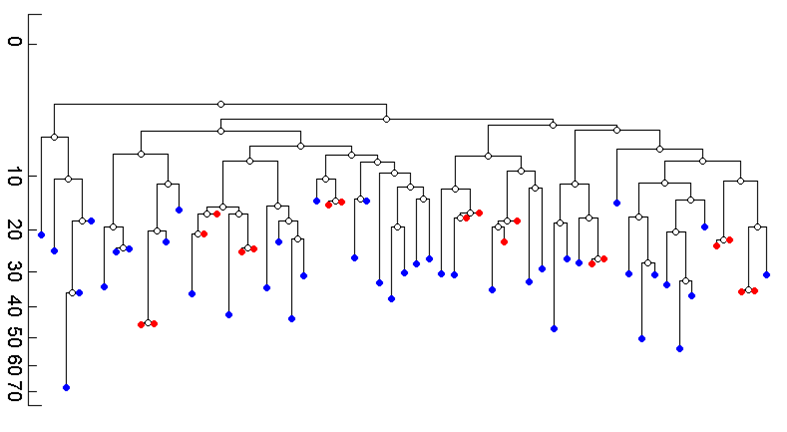

Supplement: Figure S10 — PCR repeats are clustered on the reconstructed lineage tree – each PCR plate included a pair of repeats, with the aim of controlling for the high microsatellite mutation rates that can result in mutations occurring even during the PCR process. The reconstructed lineage tree of M23–139 including PCR repeats (red) demonstrates that PCR repeats are always strongly clustered. Similar results are seen in all trees. (TIF) [file pgen.1002477.s010.tif]

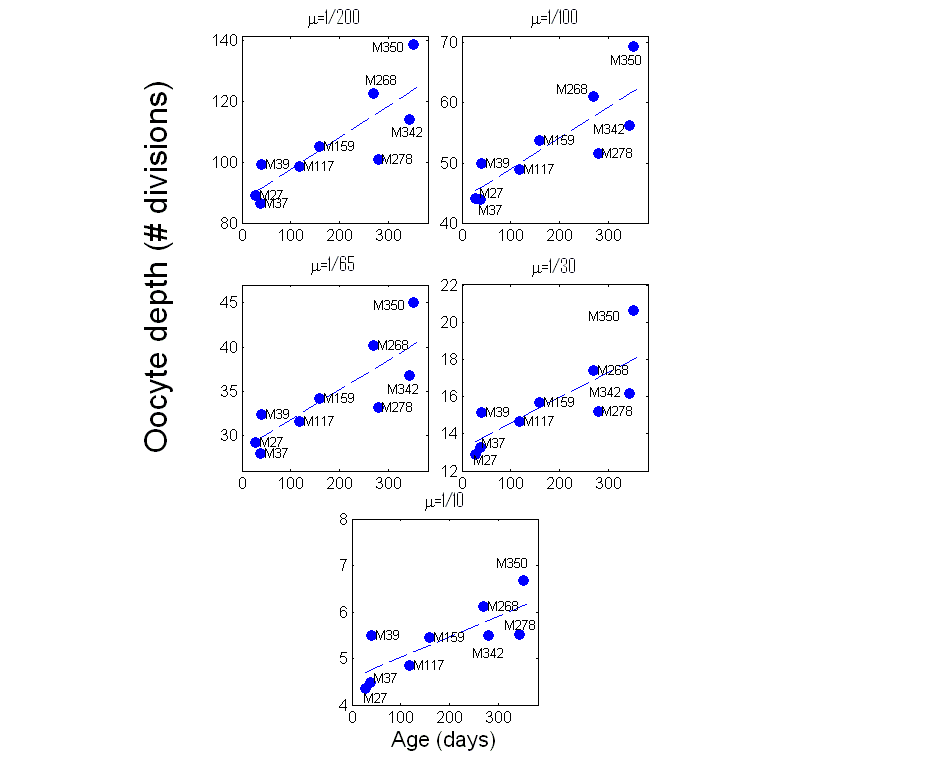

Supplement: Figure S11 — Oocyte depth increases with age using different mutation rates. Shown are five subplots, similarly to Figure 3d. The median oocyte depth of each mouse in each subplot is represented by blue circle was measured by different mutation rate, ranging from 1 mutation per 10 cell divisions to 1 mutation per 200 cell divisions. (TIF) [file pgen.1002477.s011.tif]

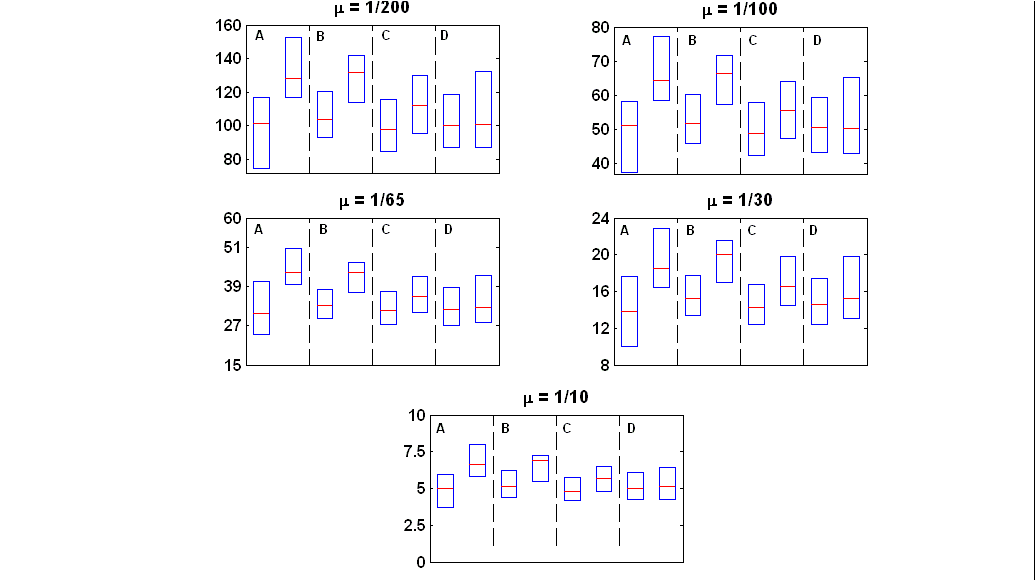

Supplement: Figure S12 — Accelerated increase in oocyte depth following unilateral ovariectomy using different mutation rates. Shown are five subplots, similarly to Figure 4d, each subplot contains 4 columns: A. M23–139 B. M29–161 C. M26–150 D. Left - M23, M26, M29. Right - M117, M159. The median of oocyte depth in each animal at each age in each subplot was measured by different mutation rate, ranging from 1 mutation per 10 cell divisions to 1 mutation per 200 cell divisions. (TIF) [file pgen.1002477.s012.tif]

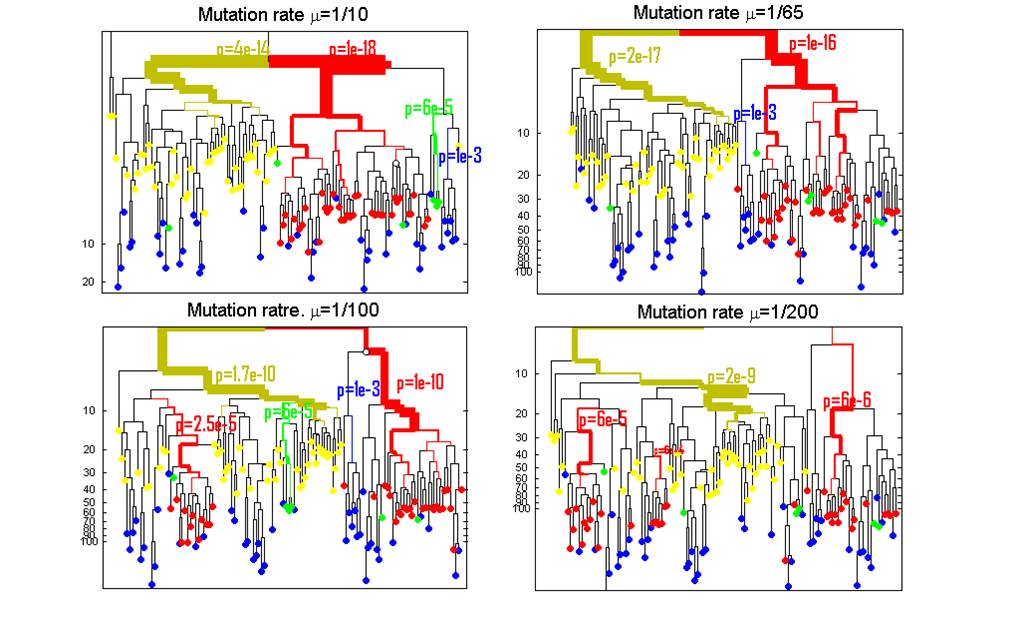

Supplement: Figure S13 — Oocytes form a cluster distinct from bone marrow cells using different mutation rates. Here we demonstrate the clustering results showed in Figure 1, are robust to variations in the mutation rate. In Figure 1 we used mutation rate of 1 mutation per 30 cell divisions in each locus. Shown is representative cell-lineage trees of M37 for a few other mutation rates in the range [1/10–1/200].It can be seen that for all the different mutation rates, oocyte are clustered separately from bone marrow cells. Oocytes are presented in red, mesenchymal stem cells in yellow, lymphocytes in blue and ovarian cumulus cells in green. Shaded boxes denote subtrees that are statistically enriched for cells of a certain cell population, using the same hyper-geometric enrichment test as we used in the main text. Bold branches represent statistical enriched clades on the lineage tree. The most significant p value is indicated. The width of the branches represents clustering significance (wider = lower p value). The Y axis represents depth in logarithmic scale. (TIF) [file pgen.1002477.s013.tif]

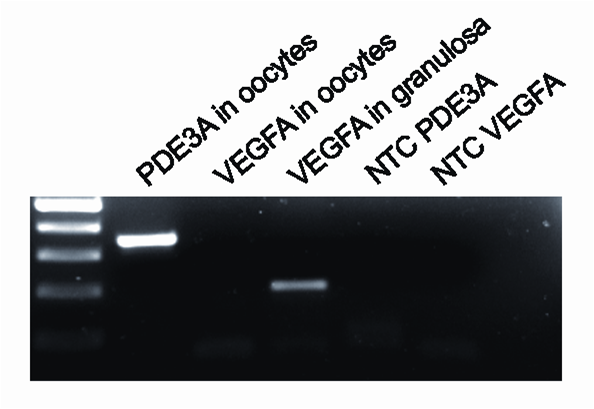

Supplement: Figure S14 — Extracted oocytes are free of granulosa contaminations. Shown are amplified PCR products of PDE3A (250 bp) in oocytes, VEGFA (100 bp) in oocytes and in granulosa cells and negative controls for both. Phosphodiesterase 3A (PDE3A), a gene highly expressed in oocytes, was used as a positive control for the quality of oocyte cDNA, and showed marked expression in this sample. (TIF) [file pgen.1002477.s014.tif]

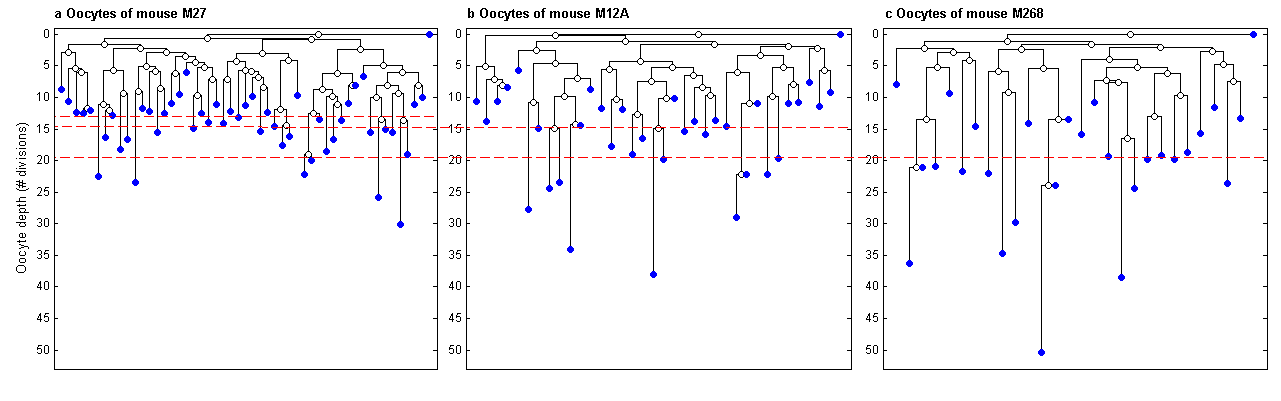

Supplement: Figure S15 — Reconstructed lineage trees of GV oocytes in three mice demonstrate oocyte depth of 12 d mouse relatively to oocyte depth in other ages (Y axis). The putative zygote is at depth 0. Median depth of oocytes is 13 divisions in M27 (a), a 27 day old mouse (Mouse names represent their age in days), 14.7 divisions in mouse M12A (b) and 19.5 in mouse M268 (c). Horizontal red lines denote the median depth. All lineage trees were reconstructed using the maximum-likelihood neighbor joining method and rooted with the median identifier of all cells. (TIF) [file pgen.1002477.s015.tif]
