# Supplementary material for: Cell Lineage Analysis of the Mammalian Female Germline
Source: PLoS Genet. 2012 Feb 23;8(2):e1002477. doi: 10.1371/journal.pgen.1002477 (PMC3285577; doi:10.1371/journal.pgen.1002477)
Supplement: Table S1 — Number and types of cells sampled from different mice. * denote wild-type mice. (DOC) [file pgen.1002477.s016.doc]

**Supplementary table 1 – Number and types of cells sampled from different mice. * denote wild-type mice.**

| **Mouse** | **Age (days)** | **Gender** | **Oocytes** | **Cumulus** | **MSC** | **Lymphocytes** | **Islet cells** | **Total** | **Panel** |
| --- | --- | --- | --- | --- | --- | --- | --- | --- | --- |
| M12A | 12 | F | 25 |  |  |  |  | 25 | A |
| M12B | 12 | F | 16 |  |  |  |  | 16 | A |
| M27 | 27 | F | 31 | 15 | 39 | 37 |  | 122 | A |
| M37 | 37 | F | 37 | 6 | 41 | 39 |  | 123 | A |
| M39 | 39 | F | 21 |  |  |  |  | 21 | A |
| M117 | 117 | F | 38 |  |  |  |  | 38 | A |
| M159 | 159 | F | 31 |  |  |  |  | 31 | A |
| M268 | 268 | F | 25 | 3 |  | 52 |  | 80 | A |
| M278 | 278 | F | 31 | 33 | 21 | 26 |  | 111 | A |
| M342 | 342 | F | 38 |  |  |  |  | 38 | A |
| M350 | 350 | F | 8 |  |  |  |  | 8 | A |
| M377* | 377 | F | 16 |  |  |  |  | 16 | A |
| M**23**-139 | 23 | F | 31 |  |  |  |  | 31 | A |
| M23-**139** | 139 | F | 16 |  |  |  |  | 16 | A |
| M**29**-161 | 29 | F | 41 |  |  |  |  | 41 | B |
| M29-**161** | 161 | F | 24 |  |  |  |  | 24 | B |
| M**26**-150 | 26 | F | 33 |  |  |  |  | 33 | B |
| M26-**150** | 150 | F | 31 |  |  |  |  | 31 | B |
| M36 | 36 | M |  |  |  |  | 12 | 8 | A |
| M280 | 280 | M |  |  |  | 0 | 25 | 76 | A |
| Total |  |  | 617 | 57 | 101 | 154 | 37 | 998 |  |
|  |  |  |  |  |  |  |  |  |  |
|  |  |  |  |  |  |  |  |  |  |
|  |  |  |  |  |  |  |  |  |  |
