# Supplementary material for: Cell Lineage Analysis of the Mammalian Female Germline
Source: PLoS Genet. 2012 Feb 23;8(2):e1002477. doi: 10.1371/journal.pgen.1002477 (PMC3285577; doi:10.1371/journal.pgen.1002477)
Supplement: Table S2 — Microsatellite loci used for lineage analysis. * denote loci that were used in the Whole genome amplification control experiment. (DOC) [file pgen.1002477.s017.doc]

**Supplementary table 2 – Microsatellite loci used for lineage analysis (^*^ denote loci that were used in the Whole genome amplification control experiment)**

Panel A

| **Locus name** | **Chromosome** | **Location** | **Basic unit** | **Number of repeats** |
| --- | --- | --- | --- | --- |
| mX3_AC30 | chrX | 160836250-160836310 | AC | 30 |
| mX8_GA35 | chrX | 156721861-156721931 | GA | 35 |
| mX31_TC35 | chrX | 62724829-62724898 | TC | 35 |
| mX64_TC36 | chrX | 10493769-10493841 | TC | 36 |
| X20**^*^** | chrX | 141974079-141974495 | T | 42 |
| mX13_A45 | chrX | 104286439-104286484 | A | 45 |
| L9348**^*^** | chr18 | 73049445-73049774 | AAG | 41 |
| mX65_TC40 | chrX | 55514979-55515058 | TC | 40 |
| mX122_TC32 | chrX | 84571609-84571673 | TC | 32 |
| mX44_A43 | chrX | 60245045-60245088 | A | 43 |
| IDT13 | chr2 | 122759810-122759969 | AC | 33 |
| X88**^v^** | chrX | 157326962-157327410 | A | 32 |
| mX73_AG30 | chrX | 91434260-91434319 | AG | 30 |
| mX54_TC32 | chrX | 55517972-55518036 | TC | 32 |
| mX56_CA30 | chrX | 6479514-6479574 | CA | 30 |
| mX46_GA29 | chrX | 69753518-69753576 | GA | 29 |
| mX39_GA30 | chrX | 93849603-93849663 | GA | 30 |
| mX36_T34 | chrX | 33177010-33177044 | T | 34 |
| mX95_AC30 | chrX | 162625830-162625889 | AC | 30 |
| mX175_AG33 | chrX | 160865087-160865152 | AG | 33 |
| mX88_GT29 | chrX | 103037800-103037858 | GT | 29 |
| mX199_T61 | chrX | 41326760-41326821 | T | 61 |
| mX133_T43 | chrX | 128906327-128906370 | T | 43 |
| mX121_A39 | chrX | 126010466-126010505 | A | 39 |
| mX187_TG30 | chrX | 110827290-110827350 | TG | 30 |
| mX138_AG32 | chrX | 35269144-35269207 | AG | 32 |
| mX149_A47 | chrX | 124841008-124841055 | A | 47 |
| mX191_TC33 | chrX | 125095762-125095827 | TC | 33 |
| mX120_T32 | chrX | 55155138-55155170 | T | 32 |
| mX141_T33 | chrX | 126673284-126673317 | T | 33 |
| M7 | chr6 | 10712484-10712733 | AAG | 36 |
| mX59_A39 | chrX | 150165449-150165488 | A | 39 |
| mX152_A38 | chrX | 67835921-67835959 | A | 38 |
| mX209_A53 | chrX | 104493618-104493671 | A | 53 |
| mX168_TC34 | chrX | 116687332-116687399 | TC | 34 |
| mX154_TC31 | chrX | 108989661-108989722 | TC | 31 |
| X68 | chrX | 127271846-127272284 | A | 35 |
| mX169_T37 | chrX | 163985269-163985306 | T | 37 |
| X51 | chrX | 112816523-112816731 | A | 43 |
| mX188_T35 | chrX | 72338589-72338624 | T | 35 |
| mX146_T37 | chrX | 159252513-159252550 | T | 37 |
| mX119_TC29 | chrX | 22314720-22314778 | TC | 29 |
| IDT9 | chr13 | 50374281-50374406 | TG | 32 |
| IDT7 | chr13 | 39218955-39219213 | AG | 32 |
| X63**^*^** | chrX | 103118981-103119292 | T | 37 |
| D1Mit1001**^*^** | chr1 | 130875104-130875234 | GT | 20 |
| X62**^*^** | chrX | 78748278-78748499 | A | 34 |
| X52**^*^** | chrX | 98363785-98364085 | T | 34 |
| X77**^*^** | chrX | 12675858-12675987 | A | 39 |
| X67 | chrX | 53549909-53550217 | A | 32 |
| IDT15**^*^** | chr1 | 72678596-72678946 | GT | 33 |
| L3464**^*^** | chr16 | 35905865-35906054 | AAG | 43 |
| IDT8 | chr6 | 141008171-141008579 | GA | 33 |
| M13**^*^** | chr14 | 85479950-85480209 | CTTT | 31 |

Panel B – extension of panel A

| Locus name | Chromosome | Location | Basic unit | Number of repeats |
| --- | --- | --- | --- | --- |
| ADR-1 | chr5 | 34161072-34161121 | T | 50 |
| ADR-38 | chr1 | 97608456-97608539 | AG | 42 |
| ADR-49 | chr7 | 109033396-109033442 | A | 47 |
| ADR-74 | chr5 | 88209875-88209919 | A | 45 |
| ADR-42 | chr2 | 66367744-66367833 | AG | 45 |
| ADR-58 | chr5 | 125442406-125442457 | T | 52 |
| ADR-94 | chr6 | 17294922-17295003 | AG | 41 |
| ADR-50 | chr1 | 104832794-104832842 | T | 49 |
| ADR-6 | chr1 | 101686725-101686777 | T | 53 |
| ADR-28 | chr7 | 3812787-3812837 | A | 47 |
| ADR-3 | chr1 | 3812787-3812837 | T | 51 |
| ADR-4 | chr5 | 120883169-120883219 | T | 51 |
| ADR-30 | chr4 | 139515332-139515379 | T | 48 |
| ADR-24 | chr5 | 118294947-118295046 | CT | 50 |
| ADR-36 | chr6 | 5083581-5083632 | T | 52 |
| ADR-70 | chr4 | 139259880-139259983 | AG | 52 |
| ADR-96 | chr7 | 6583531-6583622 | CT | 46 |
| ADR-45 | chr4 | 133622091-133622184 | CT | 47 |
| ADR-81 | chr7 | 99551139-99551188 | T | 50 |
| ADR-32 | chr1 | 64202322-64202372 | T | 51 |
| ADR-91 | chr1 | 172891809-172891890 | AG | 41 |
| ADR-27 | chr7 | 80916783-80916829 | T | 47 |
| ADR-51 | chr2 | 61972083-61972131 | A | 49 |
| ADR-5 | chr2 | 37045703-37045754 | A | 52 |
| ADR-23 | chr7 | 86641365-86641458 | CT | 47 |
| ADR-34 | chr5 | 16670882-16670932 | A | 51 |
| ADR-25 | chr2 | 117327178-117327224 | T | 47 |
