# Supplementary material for: Cell Lineage Analysis of the Mammalian Female Germline
Source: PLoS Genet. 2012 Feb 23;8(2):e1002477. doi: 10.1371/journal.pgen.1002477 (PMC3285577; doi:10.1371/journal.pgen.1002477)
Supplement: Table S3 — All oocytes extracted from the oviducts in the two cycling mice show polar bodies. (DOC) [file pgen.1002477.s018.doc]

**Supplementary table 3 – All oocytes extracted from the oviducts in the two cycling mice show polar bodies**

|  | Number of oocytes from ovaries | Number of oocytes from oviducts | Number of oviduct oocytes with polar body |
| --- | --- | --- | --- |
| M26-**150** | 24 | 7 | 7 |
| M268 | 16 | 9 | 9 |
