# Supplementary material for: Cell Lineage Analysis of the Mammalian Female Germline
Source: PLoS Genet. 2012 Feb 23;8(2):e1002477. doi: 10.1371/journal.pgen.1002477 (PMC3285577; doi:10.1371/journal.pgen.1002477)
Supplement: Text S1 — Ex-vivo calibration experiment. (DOCX) [file pgen.1002477.s019.docx]

**Ex-vivo calibration experiment**

To calibrate the mutation rate of our microsatellite panel we measured the mutations accumulated in an ex-vivo cultured cell tree, in which the depth of cells was known, as in (Wasserstrom et al, 2008). Mlh1−/− MEF cells (obtained from Michael Liskay, OHSU) were grown in medium composed of DMEM low glucose (Gibco) supplemented with 10% Fetal Bovine Serum, 1% Non-essential amino acids, and Gentamycin (70 µg/ml). Initially, a single cell was isolated from a cell stock and was defined as the tree root. This cell was allowed to proliferate for 30 cell divisions (passages were performed when required). Then, eight single cells were isolated from the root progeny, and were defined as clonal progenitors. Each clonal progenitor was expanded for 10 divisions and between 8-14 single progeny cells were sampled from each progenitor. This resulted in 90 branches leading from a progenitor to a progeny, each having undergone 10 divisions. The DNA of each single cell progeny was processed as described above. Mutation rate for each locus was estimated using maximum likelihood estimation under the assumption of a single step wise mutation model (equation 3). Average estimated mutation rate was 1/30 divisions.

**Supplementary references**

Wasserstrom A, Frumkin D, Adar R, Itzkovitz S, Stern T, Kaplan S, Shefer G, Shur I, Zangi L, Reizel Y, Harmelin A, Dor Y, Dekel N, Reisner Y, Benayahu D, Tzahor E, Segal E, Shapiro E (2008) Estimating cell depth from somatic mutations. *PLoS Comput Biol* **4:** e1000058
