# Supplementary material for: Cell Lineage Analysis of the Mammalian Female Germline
Source: PLoS Genet. 2012 Feb 23;8(2):e1002477. doi: 10.1371/journal.pgen.1002477 (PMC3285577; doi:10.1371/journal.pgen.1002477)
Supplement: Text S2 — Lineage clustering provides limits on the number of progenitors of a sampled cell population. (DOCX) [file pgen.1002477.s020.docx]

**Lineage clustering provides limits on the number of progenitors of a sampled cell population.**

In this section we demonstrate that lineage clustering can enable the estimation of the number of progenitors of a cell population. We propose a simple model of differentiation of cells from a population *P* (Figure S2). The organism lineage tree is assumed to be a binary tree, where the root at generation 0 is the zygote. At a given developmental stage – generation *T*, a group of *N* progenitors become lineage restricted, so that all of their descendants will become cells of *P*. The cell population is thus a polyclonal population – a collection of *N* clones. We assume these *N* progenitors are randomly distributed on the lineage tree. This can be the case if differentiation is caused by a localized morphogen gradient affecting several physically close cells and if the cells rapidly mix and migrate in the early embryo, so that physically close cells are not necessarily close on the lineage tree(Labouesse & Mango, 1999).

At generation *D* we sample *s* cells from *P* and additional *s* cells from the remaining extant cells and examine the lineage tree induced by these sampled cells. Lineage clustering is detected directly from the reconstructed tree if branches exist that are statistically enriched for cells of *P* using a hypergeometric test (Figure S2).

We find that for a fixed number of cells sampled the larger the number of progenitors the smaller the probability to detect lineage clustering (Figure S2).

The number of progenitors of oocytes has been estimated to range between 3 and 40(Lawson & Hage, 1994; McLaren & Lawson, 2005). We assessed the fraction of reconstructed lineage trees in which clustering was observed for different sample sizes of oocytes. Our results indicate that the number of progenitors ranges from 3-10 (Figure S3).

**Supplementary references**

Labouesse M, Mango SE (1999) Patterning the C. elegans embryo: moving beyond the cell lineage. *Trends Genet* **15:** 307-313
