# Supplementary material for: Cell Lineage Analysis of the Mammalian Female Germline
Source: PLoS Genet. 2012 Feb 23;8(2):e1002477. doi: 10.1371/journal.pgen.1002477 (PMC3285577; doi:10.1371/journal.pgen.1002477)
Supplement: Text S3 — Experimental controls. (DOCX) [file pgen.1002477.s021.docx]

**Experimental controls**

To control for the amount of mutations introduced in the whole genome amplification procedure we analyzed 10 single cells from two clones of a non-mismatch repair deficient cell line as described in(Wasserstrom et al, 2008). Each clone was expanded ex-vivo from a single cell for less than four divisions. Whole genome amplification products were analyzed over a set of representative loci showing clear allele separation (Table S2). The average squared difference in allele sizes between pairs of cells was 0.14 (standard error 0.01). This includes noise introduced during the PCR process. The average squared difference in allele sizes between PCR repeats was 0.15 (standard error 0.03). Thus PCR noise dominates over whole genome amplification introduced noise. The average squared difference in allele sizes between all pairs of samples was 1.25 (standard error 0.1). Thus the in-vivo signal is 6-times higher than the combined noise introduced by the whole genome amplification and PCR procedures.

**Supplementary references**

Wasserstrom A, Frumkin D, Adar R, Itzkovitz S, Stern T, Kaplan S, Shefer G, Shur I, Zangi L, Reizel Y, Harmelin A, Dor Y, Dekel N, Reisner Y, Benayahu D, Tzahor E, Segal E, Shapiro E (2008) Estimating cell depth from somatic mutations. *PLoS Comput Biol* **4:** e1000058
