# Supplementary material for: Cell Lineage Analysis of the Mammalian Female Germline
Source: PLoS Genet. 2012 Feb 23;8(2):e1002477. doi: 10.1371/journal.pgen.1002477 (PMC3285577; doi:10.1371/journal.pgen.1002477)
Supplement: Text S4 — Contamination control experiment. (DOCX) [file pgen.1002477.s022.docx]

**Contamination control experiment (Figure S13)**

To confirm that the oocytes were not contaminated with cumulus or granulosa cells, extracted oocytes were examined for the mRNA expression of vascular epithelial growth factor A (VEGFA), a gene that is expressed in granulosa cells but absent from oocytes.

Liquid nitrogen frozen purified oocytes and granulose cells were homogenized in 500ul Tri-Reagent (Sigma, Aldrich Corporation, St Louis, Mo, USA). Glycogen (10ul) (Roche Applied Science (Mannheim, Germany)) was added in order to allow better precipitation of RNA. After adding 100ul of chloroform to allow phase separation by centrifugation (at 4oC), 250ul of ispropanol was added to the aqueous phase. After several hours in -20oC, the RNA was precipitated by centrifugation. The pellet was washed in cold 70% ethanol and then dried. The RNA pellet was resuspended in 17ul DNase/RNase free water and DNase treatment was performed according to manufacturer's instructions (Ambion, Austin, TX, USA). Quality and quantity of total RNA extracted was assessed using Nanodrop spectrophotometer. RNA samples (200ng) were reverse-transcribed using high capacity reverse transcription kit (Applied Biosystems, Foster City, CA) as indicated in the manufacturer’s protocol. The cDNA was then diluted in a 1:100 ratio.

The reversed transcribed diluted cDNA was amplified by PCR. PCR primers were chosen using primer express software (Applied Biosystems, Foster City, CA). They were checked by the NCBI-BLAST program for their specificity. Each of these primer pairs yielded only one sharp band of amplified product with the molecular weight of the desired amplicon. The nucleotide sequences of the primers for the different genes are as follows:  VEGF A forward: CTACTGCCGTCCGATTGAG, reverse: GCTTTGGTGAGGTTTGATCC. PDE3A forward: ATGGGTAGAGCGAGCTGTGT, reverse: ACCTTGTGGAGTCAGGCATC.

VEGFA was totally absent from the oocytes samples and strongly expressed in granulosa cells (Figure S11), suggesting that extracted oocyte cells do not contain cumulus or granulose contamination.
