# Supplementary material for: Cell Lineage Analysis of the Mammalian Female Germline
Source: PLoS Genet. 2012 Feb 23;8(2):e1002477. doi: 10.1371/journal.pgen.1002477 (PMC3285577; doi:10.1371/journal.pgen.1002477)
Supplement: Text S5 — Comparison of different depth estimation methods. (DOC) [file pgen.1002477.s023.doc]

**Comparison of different depth estimation methods**

In this section we compare two methods for estimating the depth of cells from microsatellite mutations – using mean square deviation in allele sizes and using maximum likelihood estimates. We show that the maximum likelihood Neighbor-Joining method provides more efficient depth estimation, and is also more robust to deviations from the symmetric step-wise model.

We estimated depth from microsatellite mutations using methods similar to those used by(Wasserstrom et al, 2008). We reconstructed the lineage relations of cells using the Neighbor-Joining method(Saitou & Nei, 1987). We compared two methods for distance estimation – the number of divisions separating two cells. The mean squared difference estimate (denoted Tree-MS) for the distance between two cells *x^i^* and *x^j^* is given by:

(1) $d(x^{i},x^{j})=\frac{1}{l}\sum_{k=1}^{l} \left(x_{k}^{i}-x_{k}^{j}\right)^{2}$

where $x_{k}^{i}$and $x_{k}^{j}$are the number of repeats in the k-th microsatellite allele of the *i*-th and *j*-th sampled cells, respectively, and $l$is the total number of alleles. Since the one-step model of microsatellite mutation is equivalent to a *random walk*(Felsenstein, 2004), $d(x^{i},x^{j})$ is on average proportionate to $\mu t$, where *t* is the number of cell divisions separating the two cells, and $\mu$is the mutation rate per cell per division.

An alternative estimator for the number of divisions separating two cells is the maximum likelihood estimator (denoted Tree-ML). The transition probability for the one-step microsatellite mutation model yields an expression for the maximum likelihood estimate of the number of divisions separating two cells(Felsenstein, 2004; Watkins, 2007). Given a mutation rate per division μ, the transition probability is the likelihood that after *t* cell divisions the repeat number of a microsatellite locus will increase by *j* repeat units. Assuming symmetric mutations (equal probability of increasing and decreasing the repeat number) and following(Felsenstein, 2004) we write the transition probability as

(2) $P(j;t)=e^{-\mu t}\sum_{k=0}^{\infty} \left(\frac{\mu t}{2}\right)^{j+2k}\frac{1}{\left(k+j\right)!k!}$

Let $d_{x^{i,j}}=\left\{\left|x_{1}^{i}-x_{1}^{j}\right|,\left|x_{2}^{i}-x_{2}^{j}\right|,...,\left|x_{l}^{i}-x_{l}^{j}\right|\right\}$ be the vector of absolute difference between number of microsatellite repeats between cells $i$ and $j$. Then, assuming equal mutation rates for all microsatellite loci, the log-likelihood of observing that particular data set is given by

(3) $\log\left(L(d_{x^{i,j}}|\mu t)\right)=\sum_{\beta=1}^{l} \log\left(e^{-\mu t}\sum_{k=0}^{\infty} \left(\frac{\mu t}{2}\right)^{x_{\beta}^{i,j}+2k}\frac{1}{\left(k+x_{\beta}^{i,j}\right)!k!}\right)$

In practice, this expression can be numerically evaluated for reasonable values of *k* and for a range of values of *t*. Then one can find the value of *t* in this range that maximizes the log-likelihood for a given microsatellite repeat number data.

The Neighbor-Joining algorithm we applied has the following stages (Felsenstein, 2004; Studier & Keppler, 1988):

1. For each cell in the sampled set compute$u_{i}=\sum_{j\neq i}^{n} \frac{D_{i,j}}{n-2}$.
   1. For the mean squared difference estimate, let $D_{i,j}=\frac{1}{l}\sum_{k=1}^{l} \left(x_{k}^{i}-x_{k}^{j}\right)^{2}$ where $x_{k}^{i}$and $x_{k}^{j}$ are the repeat number of the *k*-th microsatellite locus of the *i*-th and *j*-th cells in the sample.
   2. For the maximum likelihood estimate, let $d_{x^{i,j}}=\left\{\left|x_{1}^{i}-x_{1}^{j}\right|,\left|x_{2}^{i}-x_{2}^{j}\right|,...,\left|x_{l}^{i}-x_{l}^{j}\right|\right\}$, the absolute value of the difference in microsatellite repeat number between cells $i$ and $j$. Then$D_{i,j}=t$, where t is the number of divisions obtained by maximizing the log-likelihood function (3).
2. Choose cells $i$and $j$for which $D_{i,j}-u_{i}-u_{j}$is smallest.
3. Join cells $i$and $j$and compute the distance from each cell to the new node:

$$v_{i}=\frac{1}{2}D_{i,j}+\frac{1}{2}\left(u_{i}-u_{j}\right)$$

$v_{j}=\frac{1}{2}D_{i,j}+\frac{1}{2}\left(u_{j}-u_{i}\right)$.

1. Compute the distance between the new node, $(i,j)$ and all other cells or nodes:

$$D_{(i,j),k}=\frac{\left(D_{i,k}+D_{j,k}-D_{i,j}\right)}{2}$$

1. Repeat until only one node remains.
2. Reroot the tree using the median microsatellite repeat number of all sampled cells.

To compare these two methods we simulated random microsatellite mutations over cells of fixed depth (either 25 divisions or 45 divisions) and computed the efficiency and bias of the two depth estimators. An estimator is more efficient the smaller the variability in its values. Mutations were simulated according to the symmetric single-step model, where at every cell division a microsatellite locus *i* was assigned a small probability µ_i_ of increasing or decreasing its repeat number by one. Mutation rates were calibrated according to the mutation rate estimates obtained from mouse ex-vivo trees. Additionally, signal dropout, where the length of a microsatellite allele in a particular cell cannot be measured due to amplification failure, was incorporated into the simulations using the dropout rates of experimental data. Finally, we evaluated the estimators’ robustness to scenarios where microsatellite mutations deviate from the single-step model, and to stochastic errors in signal analysis. To measure robustness to non-single-step events, we simulated microsatellite mutations according to a two-step model(Di Rienzo et al, 1994), where the size of the step is geometrically distributed with parameter β. To test robustness to signal errors, which may occur due to noisy capillary signal or poor allele separation, for each sampled cell we randomly chose a set of loci and randomly modified the repeat numbers for these loci.

We find that the maximum likelihood depth estimator is more efficient and less biased than the mean square estimate (Figure S1a-d). In addition the method is more robust to deviations from a single-step model and to low rates of signal errors (Figure S1e-f). We therefore chose this method for all analysis performed in the paper.

**Supplementary references**

Di Rienzo A, Peterson AC, Garza JC, Valdes AM, Slatkin M, Freimer NB (1994) Mutational processes of simple-sequence repeat loci in human populations. *Proc Natl Acad Sci U S A* **91:** 3166-3170

Felsenstein J (2004) *Inferring Phylogenies*: Sinauer Associates.

Labouesse M, Mango SE (1999) Patterning the C. elegans embryo: moving beyond the cell lineage. *Trends Genet* **15:** 307-313

Lawson KA, Hage WJ (1994) Clonal analysis of the origin of primordial germ cells in the mouse. *Ciba Found Symp* **182:** 68-84; discussion 84-91

McLaren A, Lawson KA (2005) How is the mouse germ-cell lineage established? *Differentiation* **73:** 435-437

Saitou N, Nei M (1987) The neighbor-joining method: a new method for reconstructing phylogenetic trees. *Mol Biol Evol* **4:** 406-425

Studier JA, Keppler KJ (1988) A note on the neighbor-joining algorithm of Saitou and Nei. *Mol Biol Evol* **5:** 729-731

Wasserstrom A, Frumkin D, Adar R, Itzkovitz S, Stern T, Kaplan S, Shefer G, Shur I, Zangi L, Reizel Y, Harmelin A, Dor Y, Dekel N, Reisner Y, Benayahu D, Tzahor E, Segal E, Shapiro E (2008) Estimating cell depth from somatic mutations. *PLoS Comput Biol* **4:** e1000058

Watkins JC (2007) Microsatellite evolution: Markov transition functions for a suite of models. *Theor Popul Biol* **71:** 147-159
